# Capitalising on shared goals for family planning: a concordance assessment of two global initiatives using longitudinal statistical models

**DOI:** 10.1136/bmjopen-2019-031425

**Published:** 2019-11-12

**Authors:** Qingfeng Li, Jose G Rimon, Saifuddin Ahmed

**Affiliations:** 1 International Health, Johns Hopkins Bloomberg School of Public Health, Baltimore, Maryland, USA; 2 Population, Family and Reproductive Health, Johns Hopkins Bloomberg School of Public Health, Baltimore, Maryland, USA

**Keywords:** family planning, global initiatives, FP2020, % demand satisfied

## Abstract

**Objective:**

Family planning is unique among health interventions in its breadth of health, development and economic benefits. The complexity of formulating effective strategies to promote women’s and girls’ access to family planning calls for closer coordination of resources and attention from all stakeholders. Our objective was to quantify the concordance of two global initiatives: Family Planning 2020’s adding 120 million modern contraceptive users by 2020 (proposed during The London Summit 2012 by Gates Foundation) and satisfying the 75% demand for modern contraceptives by 2030 (proposed by United States Agency for International Development). A demonstration of their concordance, or lack thereof, provides an understanding of the proposed quantitative goals and helps to formulate collective strategies.

**Design and setting:**

We applied fixed effects longitudinal models to assess the convergence of the two initiatives. The implications of success in one initiative on achieving the other are simulated to illustrate their shared goals. Publicly available data on contraceptive use, unmet need and met need from national surveys are used. Extensive model validations were conducted to check and confirm models’ predictive performance.

**Results:**

Our results show that the 75% demand satisfied initiative will reach 82 million additional modern users by 2020 and 120 million by early 2023. Following FP2020’s proposed annual increase of modern contraceptive use, 9 of the 41 commitment-making countries will reach the 75% target by 2020; another 8 countries will do so by 2030. Extending FP2020’s proposed contraceptive growth to 2030 implies the achievement of the 75% target in less than half (17) of the 41 commitment-making countries.

**Conclusion:**

The results from the statistical exercise demonstrate that the two global initiatives move toward the same goal of promoting access to family planning and overall both are ambitious. Closer coordination between major stakeholders in international family planning may stimulate more efficient mobilisation and utilisation of global sources, which is urgently needed to accelerate the progress toward satisfying women’s need for family planning.

**Strengths and limitations of this study**

- This study is the first systematic comparison of two major global initiatives on family planning.
- The estimations are based on rigorously developed and validated statistical models.
- The findings provide new insights into the shared goals of the two initiatives and have important policy implications.
- Relying on secondary data restricts variable selection for the statistical models.
- The linear growth curve assumed for modern contraceptive prevalence rate in the study may not be accurate for each country.

## Introduction

Access to family planning is a critical component of reproductive rights and leads to multifaceted benefits for women and their families. It is unique among health interventions in its breadth of health, development and economic benefits, such as reducing maternal and child mortality, empowering women and girls, and enhancing environmental sustainability.^1 2^ The Lancet series on family planning in 2012 documented strong evidence of the extensive gains resulting from family planning. Ahmed and colleagues^2^ estimated that contraceptive use in 172 countries averted 272 040 maternal deaths in 2008, and satisfying unmet need for contraceptive methods could prevent another 104 000 deaths per year. Cleland and colleagues^3^ made nearly identical estimates using a different methodology. Additionally, Canning and Schultz^4^ evaluated the economic consequences of family planning, including increases in female labour force participation and proportion in paid employment.

However, after reaching their global peak following the 1994 International Conference on Population and Development (ICPD) in Cairo, both financial support and political commitments for family planning have plateaued, and even declined in many countries, in the decade prior to 2012.^1 5^ Consequently, progress towards providing access to contraception for women and girls in developing countries has been slow. On average, women in Sub-Saharan Africa continue to have more than five children.^6^

Compared with other public health interventions, family planning has two unique features that need special attention. First, due to cultural, religious and political reasons, family planning is more controversial than many other public health issues.^1^ Even the proponents of family planning disagree with each other over what the primary aims should be. Some emphasise ecological concerns, specifically the effect of fertility declines on population structure, ecosystem and economy. Others emphasise human rights concerns, promoting women’s control over their own reproduction.^7^

Second, unlike other public health issues, such as reducing child mortality, the biomedical side of family planning is well-established, with proven methods to space and limit pregnancies. Where the successful implementation of family planning programme is concerned, it has been established that a key element is a political issue of obtaining support from and forming a broad coalition of elite groups.^1 7^ This has proven successful in many countries, but remains elusive in some, especially in Sub-Saharan Africa.

The complexity of formulating effective strategies to promote women’s and girls’ access to family planning calls for closer coordination of resources and attention from all stakeholders. As noted by Kim and Ammann,^8^ a clear consensus on targets and priorities is indispensable for all successful public projects in the modern era.

During the past few years, two major family planning initiatives were launched. First, the London Summit on Family Planning in July 2012 was convened by the United Kingdom’s Department for International Development (DFID) and the Bill and Melinda Gates Foundation (BMGF). At the Summit, leaders proposed adding 120 million female modern contraceptive users in the world’s 69 poorest countries by 2020.^9^ The second initiative, led by the United States Agency for International Development (USAID), proposed a target of satisfying 75% of the demand for family planning with modern contraceptives by 2030.^10 11^ This indicator of demand satisfied was subsequently adopted in the sustainable development goals.^12^ The % demand satisfied is the proportion of women who use modern contraception divided by the total demand for family planning, which is defined by adding the percentage of married or in-union women aged 15–49 years who are using any contraception to the percentage of women with unmet need. Unmet need refers to the proportion of women who want to stop or delay childbearing but are not using any method of contraception. Following Fabic *et al*,^10^ in the present study, we only consider the demand for FP among married or in-union women aged 15–49 years.

FP2020% and 75% demand satisfied are two ambitious family planning initiatives. A recent assessment of FP2020 found that progress has been made with diverse country-level growth rates, but overall the initiative is below the proposed trajectory.^13^ Given the scale of the initiatives and the number of partners involved in the family planning field, improved coordination and a broader coalition are necessary to achieve the goals. The objective of this study is to assess the concordance of these two initiatives by estimating the implication of accomplishing one target on the other. A demonstration of their consistency, or the lack thereof, provides a better understanding of the proposed quantitative goals and helps to formulate collective strategies.

## Methods

The contraceptive prevalence data are from the United Nations Development Programme (UNDP) survey-based estimates of the percentage of married or in-union women aged 15–49 years using any modern contraceptive method.^14^ The database includes estimates of modern contraceptive prevalence rate (mCPR) and % demand satisfied collected from 466 surveys in 142 countries from 1986 to 2016. Among the 70 FP2020 focus countries (South Africa joined the FP2020 Initiative after the London Summit), three countries (Djibouti, Somalia and Western Sahara) do not have any survey-based estimates of mCPR and % demand satisfied and therefore are excluded from the present study. In the end, our study is based on 67 FP2020 countries, with a focus on the 41 countries that made a commitment to the FP2020 Initiative (defined as commitment-making countries; see www.familyplanning2020.org for a full and up-to-date list; accessed on 20 February 2019).

The target measures discussed in this study are closely correlated by definition. Let P denote the total number of women aged 15–49 years, N denote the number of women who express a need for family planning, C denote the number of female modern contraceptive users, T denote the number of modern and traditional contraceptive users, U denote the number with unmet need for family planning. Then, we have mCPR=C/P, % unmet need=U/P and % met need (or demand satisfied)=C/N.

$$\%satisfieddemand=\frac{C}{N}=\frac{C}{T+U}=\frac{mCPR}{CPR+\%unmetneed}$$

An increase in C implies higher mCPR, but it does not necessarily increase % demand satisfied. The relationship between the indicators becomes complex in other scenarios, such as when more women express a need for family planning. This will decrease the % met need without affecting mCPR. The congruence, and lack of it, has been observed in FP2020 countries. From 2012 to 2017, the high growth of mCPR has driven a 9 percentage point increase in demand satisfied in Eastern and Southern Africa. During the same period, Central and West Africa experienced comparable mCPR growth, but that was accompanied by increasing levels of unmet need. These are the results of a complex dynamic involving both fertility intentions and available family planning services. As a result, our subsequent empirical analyses will be based on probabilistic statistical regression rather than deterministic mathematical relationships.

Another complicating factor is that FP2020 counts all women, irrespective of their marital status, while the 75% target only covers married or in-union women. Although subsequent debates consider expanding the demand satisfied target to all women, no consensus has been reached, and therefore, we will use the original statement of the 75% target. The difference in denominators will be dealt with in our statistical models.

**Figure 1:**
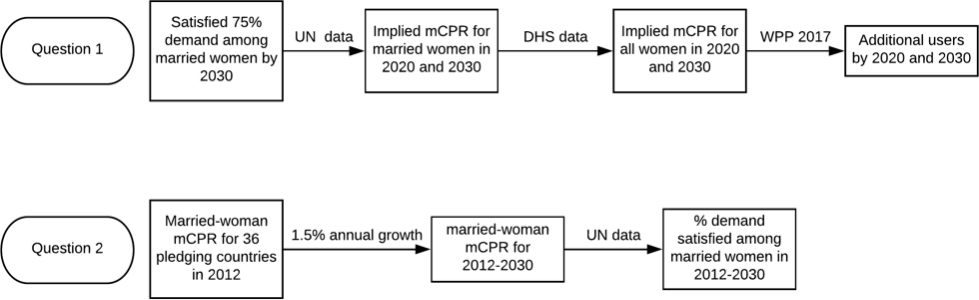
Analytical flowchart for the two research questions. mCPR, modern contraceptive prevalence rate. (DHS: Demographic and Health Surveys; WPP: World Population Prospects)

The congruence between FP2020% and 75% demand satisfied targets requires a bi-directional assessment. We estimated the implications of achieving one of them on the other. Specifically, the study attempts to answer the following two questions: (1) how many additional users will be added following the 75% demand satisfied target; (2) what percentage of demand will be satisfied in 41 commitment-making countries assuming an annual increase of 1.4 percentage points from 2012 until 2030? Annual growth of 1.4% is the overall target proposed by the London Summit on Family Planning Metrics Group across all FP2020 focus countries.^9^ Overall annual growth of 0.7 percentage points was observed across the world’s 69 poorest countries before 2012. Brown *et al* 9 estimated that doubling the annual growth to 1.4 would add 120 million female modern contraceptive users by 2020. The target growth rate is considered an aspirational yet achievable goal assuming that the resources and leadership around current family planning programme may be collectively mobilised. These two assessments are conducted separately, although employing a similar methodology (figure 1).

There are three steps to answer the first research question. The first step is to estimate the necessary married-woman mCPR to satisfy 75% demand with modern methods by 2030. Among the 41 commitment-making countries, five FP2020 commitment-making countries had already reached the 75% demand satisfied goal in their most recent surveys (table 1). It is reasonable to assume that maintaining at least 75% demand satisfied by 2030 is the goal in those countries. We assume that the mCPR and % demand satisfied will remain at their most recent observed level until 2030.

**Table 1:** Modern contraceptive prevalence rate (mCPR) in five commitment-making countries where % demand satisfied exceeded 75% in the last survey

Country	Region	Survey date	mCPR	% demand satisfied
Myanmar	Non-SSA	2015–2016	51.3	75.0
Kenya	SSA	2015	62.6	76.2
Indonesia	Non-SSA	2015–2016	59.5	78.8
South Africa	SSA	2003–2004	59.8	81.1
Zimbabwe	SSA	2015	65.8	85.2

Non-SSA includes all other regions.

SSA, sub-Sahara Africa.

For the other 36 countries, the percentage of demand satisfied with modern methods is assumed to reach 75% in 2030. Then, we employ the following country-level fixed effects longitudinal model to estimate the required mCPR for the assumed 75% demand satisfied.

(1) $$y_{it}=\beta_{0}+\beta_{1}x_{it}+\beta_{2}x_{it}^{2}+\alpha_{i}+\varepsilon_{it}$$

where $y_{it}$ denotes the mCPR for country i in time t; $x_{it}$ denotes the % demand satisfied for country i in time t; $\alpha_{i}$ denotes the time-invariant unobserved fixed effects for country i; $\varepsilon_{it}$ denotes the error term. The mode is chosen from several options due to its best predictive performance. The model is first fitted using survey-based data compiled by the UN. The least squares dummy variable method is used in the model estimation.^15^ This approach explicitly provides the coefficients of the country dummy, which is required in predicting the mCPR for the assumed 75% demand satisfied in 2030. Then with the estimated coefficients and country-level fixed effects, we estimate mCPR for the assumed 75% demand satisfied.

The second step is to convert the married-woman mCPR estimated in step 1 to all-woman mCPR. Two hundred sixty-two Demographic and Health Surveys (DHS) based on samples of all women of reproductive ages were conducted from 1990 to 2016 in 85 countries. We use the following fixed effects longitudinal model to estimate all-woman mCPR from married mCPR:

(2) $$a_{i}=\theta_{0}+\theta_{1}m_{i}+v_{i}+\epsilon_{it}$$

where $a_{i}$ and $m_{i}$ denote the all-woman and married mCPR in survey i; $v_{i}$ denotes region (SSA vs non-SSA) level fixed effects. We use region level instead of country-level fixed effects because a model with country-level fixed effects cannot be used for prediction in FP2020 countries without a DHS survey.

In the third step, we assume that all-woman mCPR will increase linearly from the level in the last survey to the level estimated for 2030 in step 2. Using the number of women of reproductive age obtained from World Population Prospects 2017, we calculate the number of female modern contraceptive users in the 67 FP2020 focus countries.^16^

The second research question is answered similarly in three steps (figure 1). We first estimate the baseline, that is, all-woman mCPR in 2012. Our principle is to rely on the survey-based estimates as much as possible. As mentioned above, 5 of the 41 FP2020 commitment-making countries had already reached the 75% demand satisfied demand goal in their most recent surveys and therefore are excluded from this investigation. Among the other 36 commitment-making countries, 10 conducted a survey in 2012. For those 19 countries that have conducted surveys both before and after 2012, we use the two surveys before and after 2012 to linearly interpolate the mCPR for 2012. For the other seven countries that only have surveys conducted before 2012, we used the last survey-based estimate for 2012.

Then we impose a 1.4% annual increase in all-woman mCPR from 2012 until 2030. Finally, we predict the % demand satisfied demand associated with the calculated levels of all-woman mCPR for 2012–2030 based on a fixed effects longitudinal model similar to Equation (1), but moving % demand satisfied to the left-hand side and including mCPR and its squared term in the right-hand side.

### Patient and public involvement

The study does not involve patients or the public.

## Results

All three fixed effects longitudinal models fit the data quite well, indicating excellent predictive performance (table 2). Using 466 survey-based estimates, the adjusted R-squared of the model regressing married-woman mCPR on % demand satisfied and a country dummy is above 0.98, meaning that less than 2% of the variations in married-woman mCPR cannot be explained by the model (Model 1). As a result, the estimated married-woman mCPR based on the assumed 75% demand satisfied should be highly accurate and reliable. The adjusted R-squared of 0.97 in Model 2 also indicates accurate conversion from married-woman to all-woman mCPR. Model 3 that regresses % demand satisfied on married-woman mCPR also performed well (adjusted R-squared 0.97).

**Table 2:** Goodness of fit of the fixed effects longitudinal models

	Model 1	Model 2	Model 3
Outcome	Married mCPR	All-woman mCPR	% satisfied demand
Covariates	% demand satisfied (% demand satisfied)^∧^2	Married mCPR	Married mCPR (married mCPR)^∧^2
Fixed effects	Country level	Region (SSA; non-SSA) level	Country level
Sample size	466	262	466
R-squared	0.98	0.91	0.97

mCPR, modern contraceptive prevalence rate; SSA, sub-Sahara Africa.

**Figure 2:**
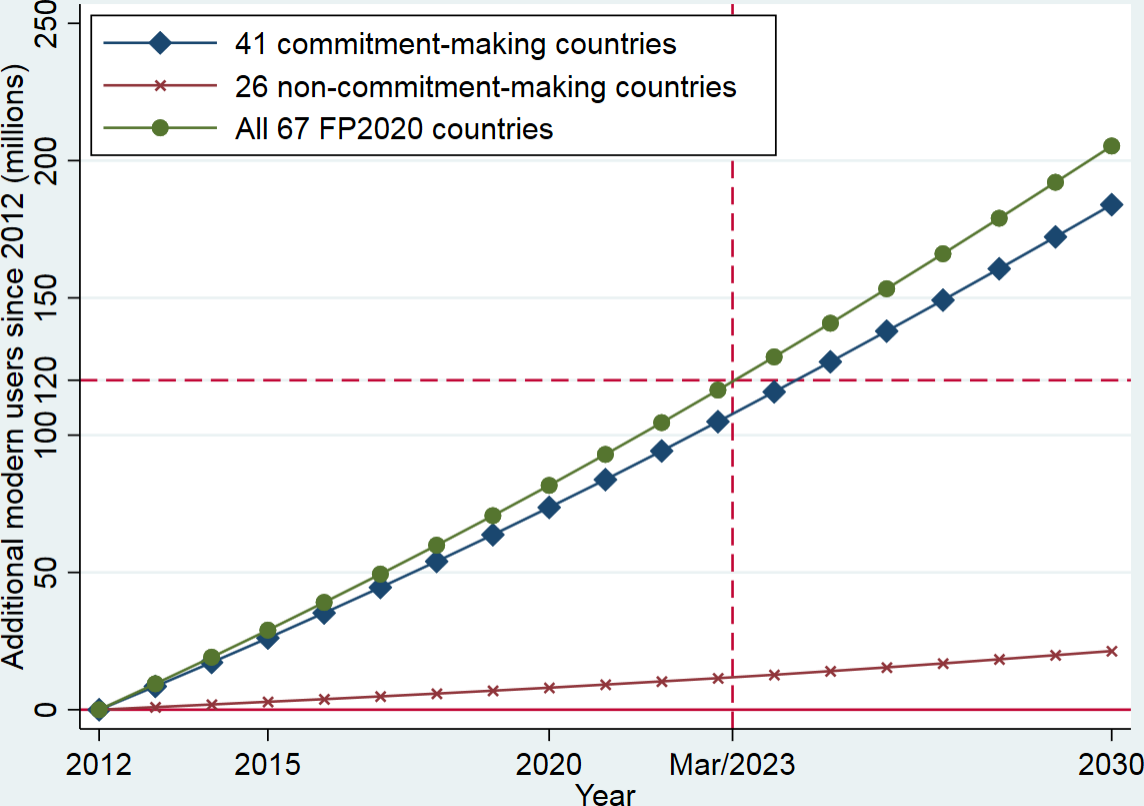
Number of additional female modern contraceptive users in 41 commitment-making and 26 non-commitment-making countries assuming the trajectory of satisfying 75% demand by 2030.

Achieving the 75% demand satisfied by 2030 goal means a gain of approximately 82 million additional users in these 67 FP2020 countries from 2012 to 2020, which is about 68% of the 120 million proposed by the FP2020 Initiative (table 3). From 2012 to 2020, these 41 commitment-making countries will contribute 74 million additional users while these 26 non-commitment-making FP2020 countries contribute 8 million. If the 67 countries continue the mCPR growth rate implied by the 75% demand satisfied initiative, the goal of adding 120 million female modern contraceptive users will be achieved in early 2023 (figure 2). By 2030, there will be 184 and 21 million additional users in commitment-making and non-commitment-making countries, respectively, making a total number of 206 additional modern contraceptive users in these 67 FP2020 countries.

**Table 3:** Modern contraceptive prevalence rate (mCPR), modern contraceptive users (thousand) and added users since 2012 (thousand) in 41 commitment-making and 26 non-commitment-making countries under FP2020: assuming the achievement of 75% demand satisfied by 2030

Country	2012		2020			2030		
	mCPR	Users	mCPR	Users	Added users	mCPR	Users	Added users
Commitment-making countries (41)								
Afghanistan	10.4	691	22.7	2065	1374	38.1	4645	3954
Bangladesh	41.2	17 800	42.4	20 200	2366	43.8	22 200	4369
Benin	7.0	158	22.0	636	478	40.9	1570	1412
Burkina Faso	13.5	515	25.5	1249	734	40.5	2704	2189
Burundi	16.7	371	27.5	768	397	41.0	1599	1229
Cameroon	14.2	708	25.8	1621	913	40.3	3362	2655
Chad	0	0	17.2	651	651	39.3	2052	2052
Côte d’Ivoire	11.5	566	24.2	1509	943	39.9	3277	2711
DR Congo	3.9	594	20.3	4066	3472	40.8	11 600	11 000
Ethiopia	26.2	5677	32.6	9285	3608	40.5	15 100	9398
Ghana	14.3	935	26.9	2093	1158	42.6	4080	3145
Guinea	4.8	124	20.1	655	531	39.2	1711	1588
Haiti	24.5	663	32.9	1009	346	43.3	1493	829
India	35.7	115 000	38.5	138 000	22 600	41.9	162 000	46 700
Indonesia	45.7	31 000	45.7	32 900	1904	45.7	34 600	3521
Kenya	47.1	5076	47.7	6592	1516	48.5	8575	3499
Laos	33.4	570	38.1	742	171	44.1	972	401
Liberia	14.2	139	25.9	318	179	40.6	649	510
Madagascar	26.2	1395	32.8	2246	851	41.0	3674	2280
Malawi	45.4	1696	44.7	2218	522	44.0	2984	1289
Mali	7.6	268	21.2	962	694	38.1	2427	2160
Mauritania	7.6	70	22.2	259	189	40.5	610	540
Mozambique	11.3	676	23.3	1792	1115	38.2	4002	3325
Myanmar	39.5	5503	39.5	5956	453	39.5	6197	694
Nepal	34.0	2504	38.1	3293	789	43.4	4078	1574
Niger	5.2	192	19.2	971	778	36.6	2781	2588
Nigeria	6.2	2364	20.5	9773	7409	38.5	24 200	21 900
Pakistan	20.0	8935	29.5	15 400	6475	41.3	26 100	17 100
Philippines	28.1	7022	35.8	10 100	3100	45.5	14 700	7664
Rwanda	36.1	974	39.2	1326	353	43.1	1880	906
Senegal	12.8	429	24.7	1040	611	39.6	2217	1787
Sierra Leone	11.6	188	23.7	479	291	38.8	997	809
Solomon Islands	24.1	33	28.0	45	13	32.7	64	31
South Africa	46.4	6715	46.4	7366	651	46.4	8067	1352
South Sudan	6.3	160	21.2	697	537	39.9	1715	1555
Togo	11.8	195	25.1	516	322	41.7	1109	915
Uganda	20.5	1652	30.7	3355	1703	43.4	6715	5062
Tanzania	21.8	2483	30.1	4459	1976	40.4	8243	5760
Vietnam	43.4	11 200	45.8	11 900	686	48.8	12 600	1401
Zambia	34.3	1176	38.0	1731	555	42.7	2627	1451
Zimbabwe	50.9	1944	50.9	2366	422	50.9	3010	1066
Subtotal		238 350		312 597	73 836		423 176	184 372
Non-commitment-making countries (26)								
Bhutan	50.2	101	50.2	114	14	50.2	124	23
Bolivia	30.5	788	37.6	1129	341	46.6	1590	801
Cambodia	28.5	1159	35.1	1581	422	43.3	2281	1121
Central African Republic	12.7	133	24.7	292	158	39.7	627	493
Comoros	12.0	21	25.3	54	33	41.9	114	92
Congo	10.9	120	24.9	334	214	42.3	755	635
Egypt	43.8	9961	43.8	11 200	1256	43.8	13 200	3262
Eritrea	9.9	108	23.1	311	203	39.6	704	595
Gambia	5.6	24	19.8	109	85	37.6	282	258
Guinea-Bissau	10.2	41	18.1	90	49	27.9	180	139
Honduras	49.0	1104	49.0	1319	215	49.0	1495	391
Iraq	28.5	2270	33.5	3367	1097	39.6	5157	2887
Kyrgyzstan	26.3	401	32.1	501	100	39.4	703	301
Lesotho	46.4	251	46.4	290	39	46.4	337	86
Mongolia	36.9	305	39.5	331	26	42.8	392	87
Nicaragua	59.2	960	59.2	1053	93	59.2	1119	159
North Korea	58.5	3899	58.5	3800	−100	58.5	3654	−246
Palestine	33.1	348	37.0	490	143	41.9	717	369
Papua New Guinea	24.6	449	32.5	717	267	42.3	1130	680
Sao Tome and Principe	27.3	12	36.2	19	7	47.4	32	20
Sri Lanka	42.1	2251	44.6	2374	123	47.6	2473	222
Sudan	6.5	552	21.1	2240	1688	39.3	5425	4873
Tajikistan	20.3	428	28.0	670	242	37.5	1086	657
Timor-Leste	18.2	46	24.0	74	28	31.1	127	81
Uzbekistan	39.9	3334	40.1	3597	263	40.3	3964	630
Yemen	21.8	1326	30.3	2339	1013	41.0	4079	2753
Subtotal		30 395		38 397	8019		51 744	21 372
Total (67 focus countries)		268 745		350 995	81 855		474 920	205 744

The predicted mCPR for Chad 2012 was rounded to 0; the columns may add up exactly because our statistical models used exact numbers while results are presented in thousands.

Five of the 41 FP2020 commitment-making countries (three in sub-Saharan Africa) have already satisfied 75% or more of the contraceptive demand, according to their last survey. Among the other 36 commitment-making countries, only four additional countries (Bangladesh, India, Malawi and Vietnam) will reach that target by 2020, following FP2020’s proposed 1.4% annual increase in mCPR (table 4). Another eight countries (Ethiopia, Laos, Madagascar, Nepal, Rwanda, Solomon Islands, Tanzania and Zambia) will do so by 2030. Disaggregated by region, the situation is more challenging in sub-Saharan Africa, where only one (Malawi) of the 26 commitment-making countries will reach the 75% target by 2020, and another five countries (Ethiopia, Madagascar, Rwanda, Tanzania and Zambia) will do so by 2030. Adding those three countries that had already reached the target in their most recent surveys, less than one-third (9) of the 29 commitment-making countries in this region will satisfy 75% demand for family planning by 2030. In the other regions, five countries will achieve the target by 2020, and another three will do so by 2030. Those eight target-achieving countries represent two-thirds of the 12 non-SSA commitment-making countries.

**Table 4:** Modern contraceptive prevalence rate (mCPR) and % demand satisfied (%SD) in 2012, 2020 and 2030 in 36 commitment-making countries: assuming the achievement of FP2020 and extending its mCPR trajectories to 2030

Country	2020		2030	
	mCPR	%SD	mCPR	%SD
Sub-Sahara Africa (26)				
Benin	19.1	33.7	33.1	52.0
Burkina Faso	27.4	47.1	41.4	63.5
Burundi	28.9	46.5	42.9	62.5
Cameroon	27.8	48.3	41.8	64.5
Chad	15.9	31.4	29.9	50.5
Côte d’Ivoire	23.7	42.6	37.7	59.8
DR Congo	18.8	33.5	32.8	51.9
Ethiopia	40.9	63.6	54.9	**76.7**
Ghana	32.8	50.5	46.8	65.6
Guinea	15.8	31.8	29.8	50.9
Liberia	28.9	49.0	42.9	65.0
Madagascar	40.4	61.4	54.4	**74.7**
Malawi	62.1	**77.7**	76.1	**85.8**
Mali	21.1	42.6	35.1	60.5
Mauritania	22.7	40.8	36.7	58.3
Mozambique	22.5	45.6	36.5	63.1
Niger	23.4	49.9	37.4	67.2
Nigeria	22.0	44.7	36.0	62.3
Rwanda	56.8	73.3	70.8	**82.6**
Senegal	27.3	48.6	41.3	65.0
Sierra Leone	24.9	47.9	38.9	64.9
South Sudan	12.9	23.8	26.9	43.6
Togo	26.9	42.8	40.9	59.3
Uganda	37.0	54.6	51.0	68.7
Tanzania	40.5	63.8	54.5	**77.0**
Zambia	53.1	72.0	67.1	**82.2**
Other regions (10)				
Afghanistan	31.0	56.9	45.0	72.4
Bangladesh	70.5	**83.1**	84.5	**89.2**
Haiti	42.5	59.7	56.5	72.5
India	59.1	**78.0**	73.1	**86.8**
Laos	53.9	70.1	67.9	**80.2**
Nepal	54.4	72.1	68.4	**82.0**
Pakistan	37.3	57.7	51.3	71.6
Philippines	48.3	62.7	62.3	74.0
Solomon Islands	38.6	73.3	52.6	**87.0**
Vietnam	69.7	**77.3**	83.7	**83.6**

Bold indicates reaching the target of satisfying 75% demand for family planning; Madagascar’s 74.7% in 2030 can be rounded to 75%.

In sum, assuming FP2020’s proposed annual growth rate in mCPR, the % satisfied will reach 75% in less than half (17) of the 41 FP2020 commitment-making countries.

## Discussion

The contribution of this study is an improved understanding of the concordance of two global family planning initiatives: FP2020’s adding 120 female modern contraceptive users by 2020 in 69 of the world’s poorest countries and USAID’s satisfying 75% demand for family planning with modern contraceptives. Our results show that the two initiatives move towards the same goal of promoting access to family planning for women and girls. Overall, both the 75% demand satisfied and the FP2020 goal are ambitious. Achieving the 75% demand satisfied goal by 2030 implies that 82 million or 68% of the 120 million target users will be added by 2020 in 67 FP2020 focus countries. The target of 120 million will be achieved by 2023, only 3 years later than the FP2020 deadline. On the contrary, achieving a 1.4% annual increase in all-woman mCPR will enable only 17 of the 41 commitment-making countries to attain the goal of 75% demand satisfied by 2030. The overall assessment should not mask the across-country variations. In some countries, it is more plausible to achieve the FP2020’s proposed annual increase of 1.4 percentage points than satisfying the 75% demand by 2030. Some other countries, however, have 75% demand satisfied or will do so by 2030 with an annual mCPR increase below 1.4 percentage points.

Capitalising the shared goals, the demonstrated concordance may facilitate building a broad coalition to promote family planning in the developing world. These two initiatives represent the objectives of two major donors to family planning.^17^ The % demand satisfied has also been adopted as an indicator of the sustainable development goals. Due to their different features and advantages, mCPR (and number of users) and % demand satisfied will continue coexisting in the international agenda for family planning. Despite their theoretical correlation, the empirical relation between the two indicators depends on other context-specific factors, such as demand generation and changes in fertility desire. As a result, an assessment of the empirical correlation between the two indicators has sustaining policy implications. A consensus goal is critical to building a broad coalition to collectively and effectively mobilise financial and political resources and capture global attention.

The simulated implications of achieving one target on the other have several policy implications, which are urgently needed as donors and stakeholders are debating about the post-FP2020 plan. First, multiple measures will continue coexisting in international family planning. The FP2020 Core Group of which the BMGF, United Kingdom’s DFID, USAID and United Nations Population Fund (UNFPA) are active may renew their commitment to adding female modern contraceptive users beyond FP2020 to the FP2030 deadline. The % demand satisfied has been adopted as an indicator, Sustainable Development Goals (SDG) 3.7.1. The 75% benchmark is being used as a proxy for the minimum definition of ‘universal access to reproductive health’ in terms of contraceptive use (SDG 3). Methodologically, our models for assessing the congruence of the two measures could be replicated as the FP2020 movement sets its goals for FP2030.

Second, our exercise sheds light on the choice between aspirational and realistic target-setting approaches. The findings show that 75% demand satisfied can be viewed in three settings: (1) countries who have already achieved the goal but whose plans involve increasing the percentage higher than the 75% benchmark (eg, Indonesia, Myanmar, Kenya, South Africa, Zimbabwe); (2) countries which are projected to likely reach the goal by 2020 and 2030 and (3) countries which will remain below the goal (24 of 41 commitment-making countries). With only 1 year left before its deadline, FP2020 has contributed to the mobilisation of global resources for family planning and has shown progress against the goal but not at the trajectory to reach 120 million more women and girls by 2020.

The third policy implication is for the choice between global and national targets. All countries in our exercise belong to low-income countries, but they still demonstrate massive diversity in terms of mCPR, desired and realised fertility, and population age structure. When setting targets in the future, donors and stakeholders need to strike a balance between simplification (global target as in FP2020) and customisation (country-specific targets as in 75% demand satisfied).

The last policy implication is on SDG. Although % demand satisfied has been adopted as an indicator (SDG 3.7.1), it has not been associated with quantitative goals. The same situation occurred to Target 5b of Millennium Development Goals (MDGs): ‘Achieve, by 2015, universal access to reproductive health’. Several studies argued that clear, measurable goals could be a focal point for coalescing political support for action.^9^ Reflecting on the lag in substantively integrating family planning into the MDGs, FP2020 proposed a quantifiable target of adding 120 million female modern contraceptive users by 2020. Adopting the target of 75% demand satisfied in SDG may help mobilise and guide resource allocation and provide a benchmark for programme advocacy. Per our simulation results, the target is achievable in certain countries and aspirational in others.

The study is not without limitations. First, despite the highly satisfactory model fit, our regressions could be theoretically improved by including other factors such as calendar time. We did not include year as a covariate because its coefficient reflects not only temporal effects but also the changing composition of countries in the database. For example, the earliest DHS surveys were mostly in Africa, while Asia was added later. So, the absence of calendar time in the model is a limitation with the database rather than our methodology. Since we are mainly interested in the predictive performance of the model, measured by the adjusted R-squared, and adding year as a covariate changed the adjusted R-squared by less than 1 percentage point, our final model did not consider calendar time. The second limitation is the linear assumption on mCPR growth. Other growth curves (such as S-shaped or logistic) may be more accurate in many countries. The 67 FP2020 countries are in different stages of mCPR growth, some experiencing a convex trajectory and some a concave trajectory. Fully accounting for country-specific curves will likely make the statistical models much more complex and less robust. We believe a linear trajectory provides an acceptable approximation for the mixture of convex and concave trajectories. Consequently, the global estimates presented in the study may not be substantially affected by the assumed linearity.

As repeatedly emphasised in the London Summit document, setting a quantitative target should not cause concern among those firmly committed to sexual and reproductive health and rights because all interventions will have women’s rights at the centre of their implementation efforts. Our assessment in this study of the congruence of major, articulated family planning initiatives aims to unite international communities into collective actions that secure women’s and girls’ access to effective contraceptive methods.

## Supplementary Material

Reviewer comments

Author's manuscript
