# Supplementary material for: Capitalising on shared goals for family planning: a concordance assessment of two global initiatives using longitudinal statistical models
Source: BMJ Open. 2019 Nov 12;9(11):e031425. doi: 10.1136/bmjopen-2019-031425 (PMC6858104; doi:10.1136/bmjopen-2019-031425)
Supplement: Author's manuscript [file bmjopen-2019-031425.draft_revisions.pdf]

BMJ Open

BMJ Open is committed to open peer review. As part of this commitment we make the peer review history of every article we publish publicly available.

When an article is published we post the peer reviewers' comments and the authors' responses online. We also post the versions of the paper that were used during peer review. These are the versions that the peer review comments apply to.

The versions of the paper that follow are the versions that were submitted during the peer review process. They are not the versions of record or the final published versions. They should not be cited or distributed as the published version of this manuscript.

BMJ Open is an open access journal and the full, final, typeset and author-corrected version of record of the manuscript is available on our site with no access controls, subscription charges or pay-per-view fees (http://bmjopen.bmj.com).

If you have any questions on BMJ Open's open peer review process please email info.bmjopen@bmj.com

BMJ Open

Building a coalition to promote family planning through shared goals: assessing the concordance of two initiatives

Journal:	*BMJ Open*
Manuscript ID	bmjopen-2019-031425
Article Type:	Research
Date Submitted by the Author:	03-May-2019
Complete List of Authors:	Li, Qingfeng; Johns Hopkins Bloomberg School of Public Health, International Health<br>Rimon, Jose; Johns Hopkins Bloomberg School of Public Health, Population, Family and Reproductive Health<br>Ahmed, Saifuddin; Johns Hopkins University, Population, family and reproductive Health; Johns Hopkins University
Keywords:	Family planning, Global initiatives, FP2020, 75% satisfied demand

SCHOLARONE™
Manuscripts

Building a coalition to promote family planning through shared goals: assessing the concordance of two initiatives

Building a coalition to promote family planning through shared goals: assessing the concordance of two initiatives

Qingfeng Li[1], Oying Rimon[1], Saifuddin Ahmed[1]

[1]Bill & Melinda Gates Institute for Population and Reproductive Health
Department of Population, Family and Reproductive Health
Johns Hopkins Bloomberg School of Public Health, Baltimore, Maryland, United States of America

Corresponding author:
Qingfeng Li, MHS, PhD
Bloomberg School of Public Health
Johns Hopkins University
615 North Wolfe Street W4033
Baltimore, MD 21205
Tel:(410) 614-4890
e-mail: qli28@jhu.edu

Keywords: Family planning, Global initiatives, FP2020, 75% satisfied demand

Word Count: Abstract: 132; Body: 2558

For peer review only - http://bmjopen.bmj.com/site/about/guidelines.xhtml

Introduction: Family planning is unique among medical interventions in the breadth of health, developmental and economic benefits. The complexity of formulating effective strategies to promote women and girls' access to family planning calls for closer coordination of resources and attention from all stakeholders

Objectives: Our goal was to assess the concordance of two global initiatives through estimating the implication of accomplishing one target on the other. A demonstration of their consistency, or the lack of it, provides a better understanding of the proposed quantitative goals and helps to formulate collective strategies.

Methods: We applied fixed effects longitudinal models to assess the convergence of two initiatives: Family Planning 2020's adding 120 million modern contraceptive users by 2020; satisfying 75% demand for modern contraceptive by 2030.

Results: Our results show that the latter initiative implies that additional modern users will reach 82 million by 2020 and then 120 million in early 2023. Among the 41 countries that have made official pledges for FP2020, five had already reached the 75% satisfied demand goal in their most recent surveys. Following FP2020's proposed annual increase of modern contraceptive use, four more countries will reach the 75% target by 2020; another nine countries will do so by 2030. On the other hand, achieving the FP2020 goal by 2020 will lay a solid foundation towards the 75% target by 2030. Extending FP2020's proposed contraceptive growth to 2030 implies the achievement of the 75% target in less than half (18) of the 41 pledging countries. The situation is

For peer review only - http://bmjopen.bmj.com/site/about/guidelines.xhtml

particularly challenging in sub-Sahara Africa, where less than one third (9) of the region's 29 pledging countries will reach the 75% target.

Conclusions: Given the shared goal of promoting access to family planning, a broad coalition needs to be formed to accomplish both initiatives.

Strengths of this study

- The first systematic comparison of two major global iniatives on family planning
- Using standard datasets of high quality
- Based on rigorously developed and validated statistical model
- Generate insights of important policy implications

Limitations of this study

- Relying on secondary data restricts the choice in variable selection for the statistical model

For peer review only

For peer review only - http://bmjopen.bmj.com/site/about/guidelines.xhtml

Introduction

Access to family planning is a critical component of reproductive rights and leads to multi-faceted benefits to women and their families. It is unique among medical interventions in the breadth of health, developmental and economic benefits, such as reducing maternal and child mortalities, empowering women and girls, and enhancing environmental sustainability [1, 2]. The Lancet series on family planning in 2012 documented strong evidence of the extensive gains resulting from family planning. Ahmed and colleagues estimated that contraceptive use in 172 countries averted 272,040 maternal deaths in 2008, and satisfying unmet need for contraceptive methods could prevent another 104,000 deaths per year [2]. Cleland and colleagues made nearly identical estimates using a different methodology [3]. Additionally, Canning and Schultz evaluated the economic consequences of family planning, including increases in female labor force participation and proportion in paid employment [4].

However, after reaching their global peak following the 1994 International Conference on Population and Development (ICPD) in Cairo, both financial support and political commitment for family planning have been insufficient and even declined in the decade prior to 2012 [1, 5]. Consequently, progress towards providing access to contraception to women and girls in developing countries has been slow, and on average women in Sub-Saharan Africa continue to have more than five children [6].

Compared with other public health interventions, family planning has two unique features that need special attention. First, due to cultural, religious, and political reasons, family planning is controversial than many other public health issues [1]. Even the proponents of family planning

For peer review only - http://bmjopen.bmj.com/site/about/guidelines.xhtml

disagree with each other over what the primary aims should be. Some emphasize ecological concerns, specifically the effect of fertility decline on population structure and economy. Others emphasize human rights concerns, promoting women's control over their own reproduction [7]. Second, unlike other public health issues, such as reducing child mortality, the biomedical side of family planning is well-established, with proven methods to space and limit pregnancies. Where the successful implementation of family planning programs is concerned, it has been established that a key element is a political issue of obtaining support from and forming a broad coalition of elite groups [1, 7]. This has proved to be more elusive.

The complexity of formulating effective strategies to promote women and girls' access to family planning calls for closer coordination of resources and attention from all stakeholders. As noted by Kim and Ammann (2004), a clear consensus on targets and priorities are indispensable for all successful public projects in the modern era [8].

During the past few years, two major family planning initiatives were launched: (1) the London Summit on Family Planning of July 2012, convened by the UK Department for International Development (DFID) and the Bill & Melinda Gates Foundation (BMDF), proposed to add 120 million modern contraceptive users in the world's 69 poorest countries by 2020 [9]; (2) United States Agency for International Development (USAID) set a target of satisfying 75% of the demand for family planning with modern contraceptives by 2030 [10]. The percentage satisfied demand is the proportion of women who use modern contraception divided by the total demand for family planning, which is defined by adding the percentage of married or in-union women aged 15-49 who are using any contraception to the percentage of women with unmet need.

For peer review only - http://bmjopen.bmj.com/site/about/guidelines.xhtml

Following Fabic et al. (Lancet 2014), in the present study, we only consider the demand for FP among married or in-union women aged 15-49 years [10].

FP2020 and 75% satisfied demand initiatives are two unprecedentedly ambitious initiatives on family planning. A recent assessment has found progress has been slow and the FP2020 goal overambitious [11]. Given the scale of the initiatives and the number of partners involved in the family planning field, improved coordination, and a broader coalition is necessary to achieve the targets. The objective of this study is to assess the concordance of these two initiatives through estimating the implication of accomplishing one target on the other. A demonstration of their consistency, or the lack of it, provides a better understanding of the proposed quantitative goals and helps to formulate collective strategies.

Data

The contraceptive prevalence data are from the United Nations Development Programme (UNDP) survey-based estimates of the percentage of married or in-union women aged 15-49 using any modern contraceptive methods [12]. 466 surveys conducted from 1986 to 2016 in 142 countries collected modern contraceptive prevalence rate (mCPR) and % satisfied demand. Among the 70 FP2020 focus countries (South Africa joined the FP2020 Initiative after the London Summit), three countries (Djibouti, Somalia, and Western Sahara) do not have any survey-based estimates on mCPR and % satisfied demand and therefore are excluded from the present study. In the end, our study is based on 67 FP2020 focus countries, with a focus on the 41 countries that made a commitment to the FP2020 Initiative (defined as pledging countries; see www.familyplanning2020.org for a full and up-to-date list; accessed on February 20, 2019).

For peer review only - http://bmjopen.bmj.com/site/about/guidelines.xhtml

Methods

The congruence between FP2020 and 75% satisfied demand targets requires a bi-directional assessment. We estimated the implications of achieving one of them on the other. Specifically, the study attempts to answer the following two questions: (1) how many additional users will be added following the 75% satisfied demand target; (2): what percentage of demand will be satisfied in 41 pledging countries assuming a 1.5% annual increase from 2012 until 2030? We used 1.5% since it is close to the overall target proposed by the London Summit on Family Planning Metrics Group across all FP2020 focus countries [9]. It is considered an aspirational yet achievable goal assuming the resources and leadership around current family planning programs may be collectively mobilized. These two assessments are conducted separately, albeit employing a similar methodology (Figure 1).

<Figure 1 about here>

There are three steps to answer the first research question. The first step is to estimate the necessary married-woman mCPR to satisfy 75% demand with modern methods by 2030. Among the 41 pledging countries, five FP2020 pledging countries had already reached the 75% satisfied demand goal in their most recent surveys (Table 1). It is reasonable to assume that in those countries no additional activities are needed to achieve the 75% satisfied demand by 2030 goal. We assume the mCPR and % satisfied demand will remain at their most recent observed level until 2030.

For peer review only - http://bmjopen.bmj.com/site/about/guidelines.xhtml

<Table 1 about here>

For the other 36 countries, the percentage of FP demand satisfied with modern methods will reach 75% in 2030. Then we employ the following country-level fixed effects longitudinal model to estimate the required mCPR for the assumed 75% satisfied demand.

$$y_{it} = \beta_0 + \beta_1 x_{it} + \beta_2 x_{it}^2 + \alpha_i + \varepsilon_{it} \quad (1)$$

where y_{it} denote the mCPR for country i in time t; x_{it} denotes the % satisfied demand for country i in time t; α_i denotes the time-invariant unobserved fixed effects for country i; ε_{it} denotes the error term. The mode is chosen from serval options due to its best predictive performance. The model is first fitted using survey-based data compiled by the United Nations. The Least Squares Dummy Variable (LSDV) method is used in model estimation [13]. This approach explicitly provides the coefficients of the country dummy, which is required in predicting the mCPR for the assumed 75% satisfied demand in 2030. Then with the estimated coefficients and country-level fixed effects, we estimate mCPR for the assumed 75% satisfied demand.

The second step is to convert the married all-woman mCPR estimated in step 1 to all women all-woman mCPR. 262 DHS surveys based on samples of all women of reproductive ages were conducted from 1990 to 2016 in 85 countries. We use the following fixed effect longitudinal model to estimate all-woman mCPR from married mCPR

For peer review only - http://bmjopen.bmj.com/site/about/guidelines.xhtml

$$y_i = \beta_0 + \beta_1 x_i + u_i + \varepsilon_{it} \quad (2)$$

where y_i and x_i denote the all-woman and married mCPR in survey i; u_i denotes region (SSA vs. non-SSA) level fixed effects. We use region level instead of country level fixed effects because a model with country level fixed effects cannot be used for prediction in FP2020 countries without a DHS survey.

In the third step, we assume all-woman mCPR will increase linearly from the level in the last survey to the level estimated for 2030 in step 2. Using the number of women of reproductive age obtained from World Population Prospects 2017 [14], we calculate the number of modern contraceptive users in the 67 FP2020 focus countries.

The second research question is answered similarly in three steps (Figure 1). We first estimate the baseline, i.e., all-woman mCPR in 2012. Our principle is to rely on the survey-based estimates as much as possible. As mentioned above, 5 of the 41 FP2020 pledging countries had already reached the 75% satisfied demand goal in their most recent surveys, and therefore are excluded from this investigation. Among the other 36 pledging countries, 10 conducted a survey in 2012. For those 19 countries that have surveyed conducted both before and after 2012, we use the two surveys before and after 2012 to linearly interpolate the mCPR for 2012. For the other 7 countries that only have surveys conducted before 2012, we used the last survey-based estimate for 2012.

For peer review only - http://bmjopen.bmj.com/site/about/guidelines.xhtml

Then we impose a 1.5% annual increase in all-woman mCPR from 2012 until 2030. Finally, we predict the % satisfied demand associated with the calculated levels of all-woman mCPR for 2012-2030 based on a fixed effects longitudinal model similar to Equation (1), but reversing the meaning of y_{it} and x_{it}: y_{it} denotes the mCPR and x_{it} denotes the % satisfied demand for country i in time t.

Patient and Public Involvement

The study does not involvd patients or public.

Results

All three fixed effects longitudinal models fit the data quite well, indicating excellent predictive performance (Table 2). Using 466 survey-based estimates, the adjusted R-squared of the model regressing all-woman mCPR on % satisfied demand and a country dummy is above 0.98, meaning that less than 2% of the variations in all-woman mCPR cannot be explained by the model (Model 1). As a result, the estimated all-woman mCPR based on the assumed 75% satisfied demand should be highly accurate and reliable. The adjusted R-squared is 0.97 in the regression model of all-woman mCPR on married mCPR and a region dummy based on 262 DHS surveys (Model 2). Such a strong correlation also indicates accurate conversion from married to all-woman mCPR. Another model, regressing % satisfied demand on marred mCPR, also achieved an R-squared of 0.97, which ensures the accuracy in estimating % satisfied demand based on the assumed mCPR (Model 3).

<Table 2 about here>

For peer review only - http://bmjopen.bmj.com/site/about/guidelines.xhtml

Achieving the 75% satisfied demand by 2030 goal means a gain of approximately 82 million additional users in these 66 FP2020 countries from 2012 to 2020, which is about 68% of the 120 million proposed by the FP2020 Initiative (Table 3). From 2012 to 2020, these 41 pledging countries will contribute 74 million additional users while these 26 non-pledging FP2020 countries contribute 18 million. Following the trajectory of increasing mCPR and % satisfied demand, the goal of adding 120 million modern contraceptive users will be achieved in early 2023 (Figure 2). By 2030, there will be 184 and 21 million additional users in pledging and non-pledging countries, respectively, making a total number of 206 additional modern contraceptive users in these 66 FP2020 countries.

<Table 3 about here>

Five of the 41 FP2020 pledging countries, three of them in sub-Sahara Africa, had already satisfied 75% or more of the contraceptive demand in their last survey. Among the other 36 pledging countries, only four additional countries (Bangladesh, India, Malawi, and Vietnam) will reach that target by 2020 following FP2020's proposed 1.5% annual increase in mCPR (Table 4). Another 9 countries (Ethiopia, Laos, Madagascar, Nepal, Philippines, Rwanda, Solomon Islands, Tanzania, and Zambia). Disaggregated by region, the situation is more challenging in sub-Sahara Africa, where only one of the 26 pledging countries will reach the 75% target by 2020, and only 6 by 2030. Adding those 3 countries that had already reached the target by their most recent surveys, less than one third (9) of the 29 pledging countries in this region will satisfy 75% demand for family planning by 2030. In other regions, 5 countries will achieve the target by

For peer review only - http://bmjopen.bmj.com/site/about/guidelines.xhtml

For peer review only

2020 and 9 will do so by 2030, representing three-quarters of the 12 non-SSA pledging countries.

In sum, the % satisfied will reach 75% in less than half (18) of the 41 FP2020 pledging countries.

<Table 4 about here>

Limitations

Despite the highly satisfactory model fit, our regressions could be theoretically improved by including other factors such as calendar time. We did not include year as a covariate because its coefficient reflects not only temporal effects but also the changing composition of countries in the database. For example, the earliest DHS surveys were mostly in Africa while Asia was added later. So the absence of calendar time in the model is a limitation with the database rather than our methodology. Since we are mainly interested in the predictive performance of the model, measured by the adjusted R-squared, and adding year as a covariate changed the adjusted R-squared by less than 1 percentage point, our final model did not consider calendar time.

Discussion

The contribution of the study is an improved understanding of the convergence of targets of two global family planning initiatives: FP2020's adding 120 modern contraceptive users by 2020 in 69 of the world's poorest countries and USAID's satisfying 75% demand for family planning with modern contraceptives. We estimate and discus the implication of reaching one target on the other. The shared goal may facilitate building a broad coalition to promote family planning in

For peer review only - http://bmjopen.bmj.com/site/about/guidelines.xhtml

For peer review only

the developing world. These two initiatives represent the objectives of two major donors of family planning [15]. A consensus goal is critical to building a broad coalition to collectively and effectively mobilize financial and political resources, and capture global attention.

Our results show that the two initiatives move towards the same goal of promoting access to family planning for women and girls. Overall, achieving the 75% satisfied demand goal by 2030 implies that 120 million modern contraceptive users will be added by 2023 in 67 FP2020 focus countries, only three years later than the original goal set by FP2020. On the other hand, achieving a 1.5% annual increase in all-woman mCPR will enable less than half of the 41 pledging countries to attain the goal of 75% satisfied demand by 2030.

As repeatedly emphasized in the London Summit document, setting a quantitative target should not cause concern among those firmly committed to sexual and reproductive health and rights because all interventions will have women's rights at the center of their implementation efforts. Our assessment in this study of the congruence of major, recently articulated family planning initiatives aims to unite international communities into collective actions that secure women's and girls' access to effective contraceptive methods.

Author contributions: QL, SA, and JR devised the study and wrote the article. QL compiled the data and led the statistical modeling and analysis.

Disclosure statement: No potential conflict of interest was reported by the authors.

Ethics and consent: Not required

Funding: Bill and Melinda Gates Foundation

References

1. Cleland J, Bernstein S, Ezeh A, Faundes A, Glasier A, Innis J. Family planning: the unfinished agenda. The Lancet. 2006;368(9549):1810-27.

For peer review only - http://bmjopen.bmj.com/site/about/guidelines.xhtml

2. Ahmed S, Li Q, Liu L, Tsui AO. Maternal deaths averted by contraceptive use: an analysis of 172 countries. The Lancet. 2012;380(9837):111-25.
3. Cleland J, Conde-Agudelo A, Peterson H, Ross J, Tsui A. Contraception and health. Lancet (London, England). 2012;380(9837):149-56.
4. Canning D, Schultz TP. The economic consequences of reproductive health and family planning. Lancet (London, England). 2012;380(9837):165-71.
5. Cohen SA. London summit puts family planning back on the agenda, offers new lease on life for millions of women and girls. Guttmacher Policy Review. 2012;15(3):20-4.
6. Ezeh AC, Bongaarts J, Mberu B. Global population trends and policy options. Lancet (London, England). 2012;380(9837):142-8.
7. Shiffman J, Quissell K. Family planning: a political issue. Lancet (London, England). 2012;380(9837):181-5.
8. Kim JY, Ammann A. Is the “3 by 5” Initiative the Best Approach to Tackling the HIV Pandemic? PLoS Medicine. 2004;1(2):e37.
9. Brown W, Druce N, Bunting J, Radloff S, Koroma D, Gupta S, et al. Developing the "120 by 20" goal for the Global FP2020 Initiative. Studies in family planning. 2014;45(1):73-84.
10. Fabic MS, Choi Y, Bongaarts J, Darroch JE, Ross JA, Stover J, et al. Meeting demand for family planning within a generation: the post-2015 agenda. Lancet (London, England). 2015;385(9981):1928-31.
11. Cahill N, Sonneveldt E, Stover J, Weinberger M, Williamson J, Wei C, et al. Modern contraceptive use, unmet need, and demand satisfied among women of reproductive age who are married or in a union in the focus countries of the Family Planning 2020 initiative: a systematic analysis using the Family Planning Estimation Tool. Lancet (London, England). 2017.
12. United Nations DoEaSA, Population Division (2017). World Contraceptive Use 2017 (POP/DB/CP/Rev2017).
13. Greene WH. Econometric analysis: Prentice Hall; 2011.
14. United Nations DoEaSA, Population Division, W. World Population Prospects: The 2017 Revision, Key Findings and Advance Tables. Working Paper No. ESA/P/WP/248. 2017.

15. IHME. Financing Global Health 2014: Shifts in Funding as the MDG Era Closes. Seattle, WA: IHME2015.

For peer review only

For peer review only - http://bmjopen.bmj.com/site/about/guidelines.xhtml

Figure 1: Analytical flowchart for the two research questions

Figure 2: Number of additional modern contraceptive users in 41 pledging and 26 non-pledging countries assuming the trajectory of satisfying 75% demand by 2030

For peer review only

For peer review only - http://bmjopen.bmj.com/site/about/guidelines.xhtml

Table 1: Modern contraceptive prevalence rate (mCPR) in 5 pledging countries where % demand satisfied exceeded 75% in the last survey

Country	Region	Survey date	mCPR	% demand satisfied
Myanmar	Non-SSA	2015-16	51.3	75.0
Kenya	SSA	2015	62.6	76.2
Indonesia	Non-SSA	2015-16	59.5	78.8
South Africa	SSA	2003-04	59.8	81.1
Zimbabwe	SSA	2015	65.8	85.2

Notes: SSA denotes sub-Sahara Africa; non-SSA includes all other regions

For peer review only

Table 2: Goodness of fit of the fixed effects longitudinal models

	Model 1	Model 2	Model 3
**Outcome**	Married mCPR	All-woman mCPR	% satisfied demand
**Covariates**	% satisfied demand	Married mCPR	Married mCPR
	(% satisfied demand)^2		(married mCPR)^2
**Fixed effects**	Country level	Region (SSA; non-SSA) level	Country level
**Sample size**	466	262	466
**R-squared**	0.98	0.91	0.97

For peer review only

For peer review only - http://bmjopen.bmj.com/site/about/guidelines.xhtml

Table 3: Modern contraceptive prevalence rate (mCPR), modern contraceptive users (thousand), and added users since 2012 (thousand) in 41 pledging and 26 non-pledging countries under FP2020

Country	2012		2020			2030		
	mCPR	Users	mCPR	Users	Added users	mCPR	Users	Added users
**Pledging countries (41)**								
Afghanistan	10.4	691	22.7	2,065	1,374	38.1	4,645	3,954
Bangladesh	41.2	17,800	42.4	20,200	2,366	43.8	22,200	4,369
Benin	7.0	158	22.0	636	478	40.9	1,570	1,412
Burkina Faso	13.5	515	25.5	1,249	734	40.5	2,704	2,189
Burundi	16.7	371	27.5	768	397	41.0	1,599	1,229
Cameroon	14.2	708	25.8	1,621	913	40.3	3,362	2,655
Chad	-0.4	(12)	17.2	639	651	39.3	2,040	2,052
Côte d'Ivoire	11.5	566	24.2	1,509	943	39.9	3,277	2,711
DR Congo	3.9	594	20.3	4,066	3,472	40.8	11,600	11,000
Ethiopia	26.2	5,677	32.6	9,285	3,608	40.5	15,100	9,398
Ghana	14.3	935	26.9	2,093	1,158	42.6	4,080	3,145
Guinea	4.8	124	20.1	655	531	39.2	1,711	1,588
Haiti	24.5	663	32.9	1,009	346	43.3	1,493	829
India	35.7	115,000	38.5	138,000	22,600	41.9	162,000	46,700
Indonesia	45.7	31,000	45.7	32,900	1,904	45.7	34,600	3,521
Kenya	47.1	5,076	47.7	6,592	1,516	48.5	8,575	3,499
Laos	33.4	570	38.1	742	171	44.1	972	401
Liberia	14.2	139	25.9	318	179	40.6	649	510
Madagascar	26.2	1,395	32.8	2,246	851	41.0	3,674	2,280
Malawi	45.4	1,696	44.7	2,218	522	44.0	2,984	1,289
Mali	7.6	268	21.2	962	694	38.1	2,427	2,160
Mauritania	7.6	70	22.2	259	189	40.5	610	540
Mozambique	11.3	676	23.3	1,792	1,115	38.2	4,002	3,325

For peer review only - http://bmjopen.bmj.com/site/about/guidelines.xhtml

For peer review only

Myanmar	39.5	5,503	39.5	5,956	453	39.5	6,197	694
Nepal	34.0	2,504	38.1	3,293	789	43.4	4,078	1,574
Niger	5.2	192	19.2	971	778	36.6	2,781	2,588
Nigeria	6.2	2,364	20.5	9,773	7,409	38.5	24,200	21,900
Pakistan	20.0	8,935	29.5	15,400	6,475	41.3	26,100	17,100
Philippines	28.1	7,022	35.8	10,100	3,100	45.5	14,700	7,664
Rwanda	36.1	974	39.2	1,326	353	43.1	1,880	906
Senegal	12.8	429	24.7	1,040	611	39.6	2,217	1,787
Sierra Leone	11.6	188	23.7	479	291	38.8	997	809
Solomon Islands	24.1	33	28.0	45	13	32.7	64	31
South Africa	46.4	6,715	46.4	7,366	651	46.4	8,067	1,352
South Sudan	6.3	160	21.2	697	537	39.9	1,715	1,555
Togo	11.8	195	25.1	516	322	41.7	1,109	915
Uganda	20.5	1,652	30.7	3,355	1,703	43.4	6,715	5,062
Tanzania	21.8	2,483	30.1	4,459	1,976	40.4	8,243	5,760
Viet Nam	43.4	11,200	45.8	11,900	686	48.8	12,600	1,401
Zambia	34.3	1,176	38.0	1,731	555	42.7	2,627	1,451
Zimbabwe	50.9	1,944	50.9	2,366	422	50.9	3,010	1,066
**Subtotal**		**238,350**		**312,597**	**73,836**		**423,176**	**184,372**
**Non-pledging countries (26)**								
Bhutan	50.2	101	50.2	114	14	50.2	124	23
Bolivia	30.5	788	37.6	1,129	341	46.6	1,590	801
Cambodia	28.5	1,159	35.1	1,581	422	43.3	2,281	1,121
Central African Republic	12.7	133	24.7	292	158	39.7	627	493
Comoros	12.0	21	25.3	54	33	41.9	114	92
Congo	10.9	120	24.9	334	214	42.3	755	635
Egypt	43.8	9,961	43.8	11,200	1,256	43.8	13,200	3,262
Eritrea	9.9	108	23.1	311	203	39.6	704	595

For peer review only

For peer review only - http://bmjopen.bmj.com/site/about/guidelines.xhtml

Gambia	5.6	24	19.8	109	85	37.6	282	258
Guinea-Bissau	10.2	41	18.1	90	49	27.9	180	139
Honduras	49.0	1,104	49.0	1,319	215	49.0	1,495	391
Iraq	28.5	2,270	33.5	3,367	1,097	39.6	5,157	2,887
Kyrgyzstan	26.3	401	32.1	501	100	39.4	703	301
Lesotho	46.4	251	46.4	290	39	46.4	337	86
Mongolia	36.9	305	39.5	331	26	42.8	392	87
Nicaragua	59.2	960	59.2	1,053	93	59.2	1,119	159
North Korea	58.5	3,899	58.5	3,800	(100)	58.5	3,654	(246)
Palestine	33.1	348	37.0	490	143	41.9	717	369
Papua New Guinea	24.6	449	32.5	717	267	42.3	1,130	680
Sao Tome and Principe	27.3	12	36.2	19	7	47.4	32	20
Sri Lanka	42.1	2,251	44.6	2,374	123	47.6	2,473	222
Sudan	6.5	552	21.1	2,240	1,688	39.3	5,425	4,873
Tajikistan	20.3	428	28.0	670	242	37.5	1,086	657
Timor-Leste	18.2	46	24.0	74	28	31.1	127	81
Uzbekistan	39.9	3,334	40.1	3,597	263	40.3	3,964	630
Yemen	21.8	1,326	30.3	2,339	1,013	41.0	4,079	2,753
**Subtotal**		**30,395**		**38,397**	**8,019**		**51,744**	**21,372**
**Total (67 focus countries)**		**268,745**		**350,995**	**81,855**		**474,920**	**205,744**

For peer review only

For peer review only - http://bmjopen.bmj.com/site/about/guidelines.xhtml

Table 4: Modern contraceptive prevalence rate (mCPR) and % demand satisfied (%SD) in 2012, 2020 and 2030 in 36 pledging countries

Country	2020		2030	
	mCPR	%SD	mCPR	%SD
**Sub-Sahara Africa(26)**				
Benin	19.9	34.8	34.9	54.1
Burkina Faso	28.2	48.1	43.2	65.3
Burundi	29.7	47.5	44.7	64.4
Cameroon	28.6	49.3	43.6	66.4
Chad	16.7	32.6	31.7	52.7
Côte d'Ivoire	24.5	43.7	39.5	61.8
DR Congo	19.6	34.6	34.6	54.0
Ethiopia	42.3	65.1	57.3	**78.7**
Ghana	33.6	51.5	48.6	67.3
Guinea	16.6	33.0	31.6	53.1
Liberia	29.7	50.0	44.7	66.8
Madagascar	41.2	62.3	56.2	**76.2**
Malawi	62.9	**78.2**	77.9	**86.6**
Mali	21.9	43.7	36.9	62.5
Mauritania	23.5	41.9	38.5	60.3
Mozambique	23.3	46.7	38.3	65.2
Niger	24.2	51.0	39.2	69.2
Nigeria	22.8	45.8	37.8	64.3
Rwanda	57.6	73.9	72.6	**83.6**
Senegal	28.1	49.6	43.1	66.8
Sierra Leone	25.7	49.0	40.7	66.8
South Sudan	13.7	25.1	28.7	45.9
Togo	27.7	43.8	42.7	61.1
Uganda	37.8	55.5	52.8	70.3
Tanzania	41.3	64.7	56.3	**78.5**
Zambia	53.9	72.7	68.9	**83.3**
**Other regions (10)**				
Afghanistan	31.8	57.9	46.8	74.1
Bangladesh	71.3	**83.6**	86.3	**89.8**
Haiti	43.3	60.5	58.3	73.9
India	59.9	**78.6**	74.9	**87.7**
Laos	54.7	70.8	69.7	**81.2**
Nepal	55.2	72.8	70.2	**83.1**
Pakistan	38.1	58.5	53.1	73.2

For peer review only

For peer review only - http://bmjopen.bmj.com/site/about/guidelines.xhtml

Philippines	49.1	63.4	64.1	**75.3**
Solomon Islands	39.4	74.2	54.4	**88.6**
Viet Nam	70.5	**77.8**	85.5	**84.2**

Note: bold indicates reaching the target of satisfying 75% demand for family planning

For peer review only

For peer review only - http://bmjopen.bmj.com/site/about/guidelines.xhtml

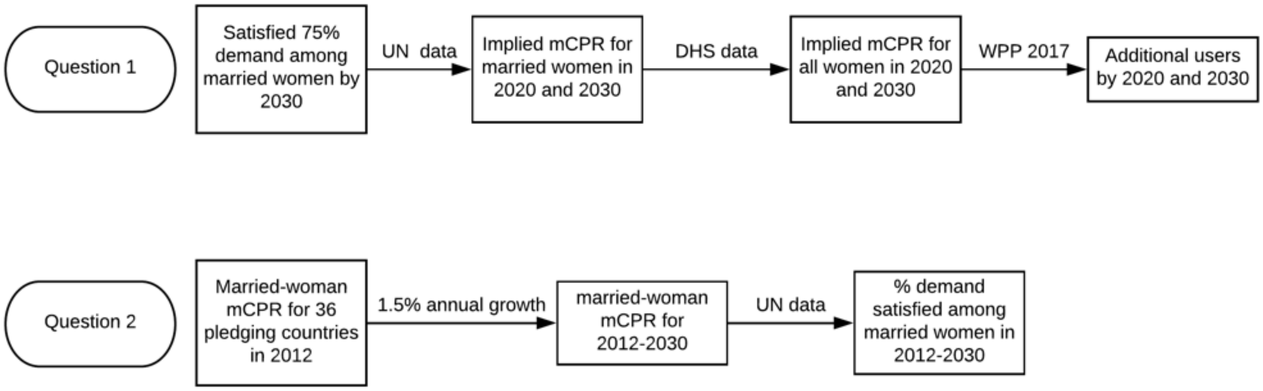
Question 1
Satisfied 75% demand among married women by 2030
UN data
Implied mCPR for married women in 2020 and 2030
DHS data
Implied mCPR for all women in 2020 and 2030
WPP 2017
Additional users by 2020 and 2030
Question 2
Married-woman mCPR for 36 pledging countries in 2012
1.5% annual growth
married-woman mCPR for 2012-2030
UN data
% demand satisfied among married women in 2012-2030

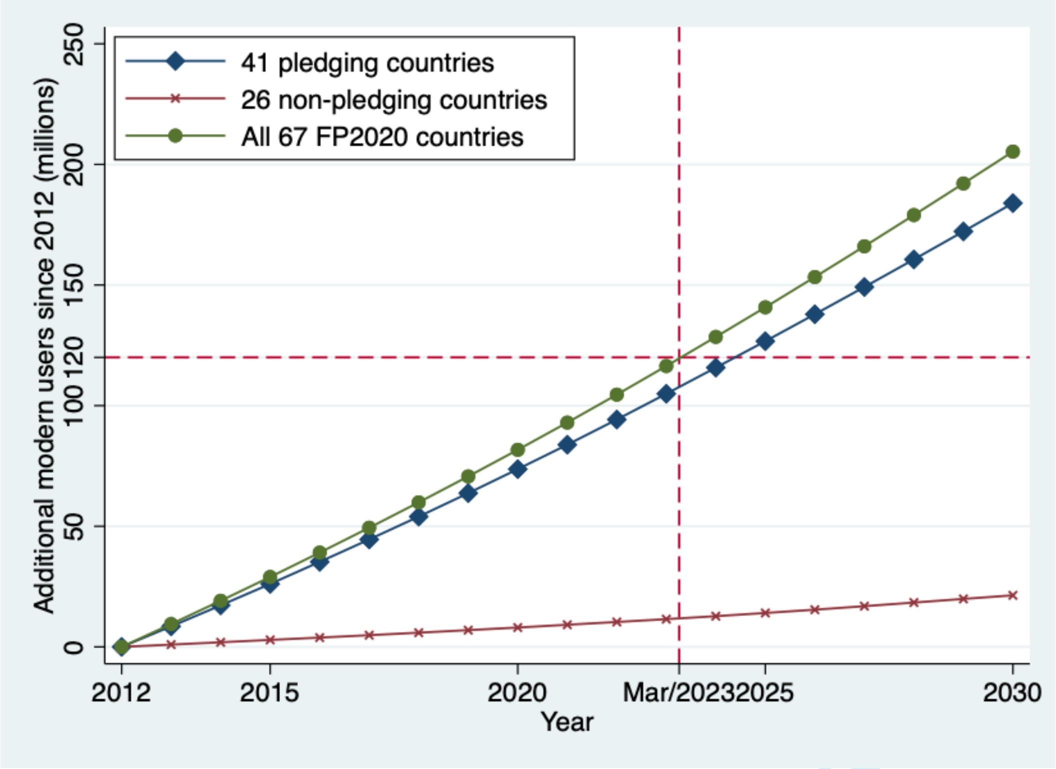
41 pledging countries
26 non-pledging countries
All 67 FP2020 countries
Additional modern users since 2012 (millions)
0
50
100
120
150
200
250
2012
2015
2020
Mar/2023
2025
2030
Year

BMJ Open

Capitalizing on shared goals for family planning: a statistical assessment of the concordance of two global initiatives

Journal:	*BMJ Open*
Manuscript ID	bmjopen-2019-031425.R1
Article Type:	Original research
Date Submitted by the Author:	12-Aug-2019
Complete List of Authors:	Li, Qingfeng; Johns Hopkins Bloomberg School of Public Health, International Health<br>Rimon, Jose; Johns Hopkins Bloomberg School of Public Health, Population, Family and Reproductive Health<br>Ahmed, Saifuddin; Johns Hopkins University, Population, family and reproductive Health; Johns Hopkins University
**Primary Subject Heading**:	Global health
Secondary Subject Heading:	Public health
Keywords:	Family planning, Global initiatives, FP2020, 75% satisfied demand

SCHOLARONE™
Manuscripts

Capitalizing on shared goals for family planning: a statistical assessment of the concordance of two global initiatives

Qingfeng Li[1], Jose G. Rimon[1], Saifuddin Ahmed[1]

[1]Bill & Melinda Gates Institute for Population and Reproductive Health
Department of Population, Family and Reproductive Health
Johns Hopkins Bloomberg School of Public Health, Baltimore, Maryland, United States of America

Corresponding author:
Qingfeng Li, MHS, PhD
Bloomberg School of Public Health
Johns Hopkins University
615 North Wolfe Street E8642
Baltimore, MD 21205
Tel:(410) 614-4890
e-mail: qli28@jhu.edu

Keywords: Family planning, Global initiatives, FP2020, 75% satisfied demand

Word Count: Abstract: 291; Body: 3555

For peer review only

For peer review only - http://bmjopen.bmj.com/site/about/guidelines.xhtml

Introduction: Family planning is unique among health interventions in its breadth of health, development and economic benefits. The complexity of formulating effective strategies to promote women and girls' access to family planning calls for closer coordination of resources and attention from all stakeholders.

Objectives: Our goal was to quantify the concordance of two global initiatives: Family Planning 2020's adding 120 million modern contraceptive users by 2020 (proposed during The London Summit 2012 by Gates Foundation) and satisfying the 75% demand for modern contraceptives by 2030 (proposed by United States Agency for International Development). A demonstration of their concordance, or lack thereof, provides an understanding of the proposed quantitative goals and helps to formulate collective strategies.

Methods: We applied fixed effects longitudinal models to assess the convergence of the two initiatives. The implications of success in one initiative on achieving the other are simulated to illustrate their shared goals. Publicly available data on contraceptive use, unmet need, and met need from national surveys are used. Extensive model validations were conducted to check and confirm models' predictive performance.

Results: Our results show that the 75% satisfied demand initiative will reach 82 million additional modern users by 2020 and 120 million by early 2023. Following FP2020's proposed annual increase of modern contraceptive use, nine of the 41 commitment-making countries will reach the 75% target by 2020; another eight countries will do so by 2030. Extending FP2020's

For peer review only - http://bmjopen.bmj.com/site/about/guidelines.xhtml

proposed contraceptive growth to 2030 implies the achievement of the 75% target in less than half (17) of the 41 commitment-making countries.

Conclusions: A closer coordination between major stakeholders in international family planning may stimulate more efficient mobilization and utilization of global sources, which is urgently needed to accelerate the progress toward satisfying women's need for family planning.

For peer review only

Strengths of this study

- This study is the first systematic comparison of two major global initiatives on family planning
- The estimations are based on rigorously developed and validated statistical models
- The findings provide new insights into the shared goals of the two initiatives and have important policy implications

Limitations of this study

- Relying on secondary data restricts variable selection for the statistical models.
- The linear mCPR growth curve assumed in the study may not be accurate for each country.

For peer review only - http://bmjopen.bmj.com/site/about/guidelines.xhtml

Introduction

Access to family planning is a critical component of reproductive rights and leads to multi-faceted benefits for women and their families. It is unique among health interventions in its breadth of health, development and economic benefits, such as reducing maternal and child mortality, empowering women and girls, and enhancing environmental sustainability.[1,2] The Lancet series on family planning in 2012 documented strong evidence of the extensive gains resulting from family planning. Ahmed and colleagues estimated that contraceptive use in 172 countries averted 272,040 maternal deaths in 2008, and satisfying unmet need for contraceptive methods could prevent another 104,000 deaths per year.[2] Cleland and colleagues made nearly identical estimates using a different methodology.[3] Additionally, Canning and Schultz evaluated the economic consequences of family planning, including increases in female labor force participation and proportion in paid employment.[4]

However, after reaching their global peak following the 1994 International Conference on Population and Development (ICPD) in Cairo, both financial support and political commitments for family planning have plateaued, and even declined in many countries, in the decade prior to 2012.[1,5] Consequently, progress towards providing access to contraception for women and girls in developing countries has been slow. On average, women in Sub-Saharan Africa continue to have more than five children.[6]

Compared with other public health interventions, family planning has two unique features that need special attention. First, due to cultural, religious, and political reasons, family planning is more controversial than many other public health issues.[1] Even the proponents of family

For peer review only - http://bmjopen.bmj.com/site/about/guidelines.xhtml

planning disagree with each other over what the primary aims should be. Some emphasize ecological concerns, specifically the effect of fertility declines on population structure, ecosystem, and economy. Others emphasize human rights concerns, promoting women's control over their own reproduction.[7]

Second, unlike other public health issues, such as reducing child mortality, the biomedical side of family planning is well-established, with proven methods to space and limit pregnancies. Where the successful implementation of family planning programs is concerned, it has been established that a key element is the political issue of obtaining support from and forming a broad coalition of elite groups.[1,7] This has proven successful in many countries, but remains elusive in some, especially in Sub-Saharan Africa.

The complexity of formulating effective strategies to promote women and girls' access to family planning calls for closer coordination of resources and attention from all stakeholders. As noted by Kim and Ammann (2004), a clear consensus on targets and priorities is indispensable for all successful public projects in the modern era.[8]

During the past few years, two major family planning initiatives were launched. First, the London Summit on Family Planning in July 2012, was convened by the United Kingdom's Department for International Development (DFID) and the Bill & Melinda Gates Foundation (BMGF). At the Summit, leaders proposed adding 120 million modern contraceptive users in the world's 69 poorest countries by 2020.[9] The second initiative, led by the United States Agency for International Development (USAID), proposed a target of satisfying 75% of the demand for

For peer review only - http://bmjopen.bmj.com/site/about/guidelines.xhtml

family planning with modern contraceptives by 2030.[10,11] This indicator of satisfied demand was subsequently adopted in the Sustainable Development Goals.[12] The percent satisfied demand is the proportion of women who use modern contraception divided by the total demand for family planning, which is defined by adding the percentage of married or in-union women aged 15-49 who are using any contraception to the percentage of women with unmet need. Unmet need refers to the proportion of women who want to stop or delay childbearing but are not using any method of contraception. Following Fabic et al., in the present study, we only consider the demand for FP among married or in-union women aged 15-49 years.[10]

FP2020 and 75% satisfied demand are two ambitious family planning initiatives. A recent assessment of FP2020 found that progress has been made with diverse country-level growth rates, but overall the initiative is below the proposed trajectory.[13] Given the scale of the initiatives and the number of partners involved in the family planning field, improved coordination, and a broader coalition is necessary to achieve the goals. The objective of this study is to assess the concordance of these two initiatives through estimating the implication of accomplishing one target on the other. A demonstration of their consistency, or the lack thereof, provides a better understanding of the proposed quantitative goals and helps to formulate collective strategies.

Methods

The contraceptive prevalence data are from the United Nations Development Programme (UNDP) survey-based estimates of the percentage of married or in-union women aged 15-49 using any modern contraceptive method.[14] The database includes estimates of modern

For peer review only

For peer review only - http://bmjopen.bmj.com/site/about/guidelines.xhtml

contraceptive prevalence rate (mCPR) and % satisfied demand collected from 466 surveys in 142 countries from 1986 to 2016. Among the 70 FP2020 focus countries (South Africa joined the FP2020 Initiative after the London Summit), three countries (Djibouti, Somalia, and Western Sahara) do not have any survey-based estimates of mCPR and % satisfied demand and therefore are excluded from the present study. In the end, our study is based on 67 FP2020 countries, with a focus on the 41 countries that made a commitment to the FP2020 Initiative (defined as commitment-making countries; see www.familyplanning2020.org for a full and up-to-date list; accessed on February 20, 2019).

The target measures discussed in this study are closely correlated by definition. Let P denote the total number of women aged 15-49 years, N denote the number of women who express a need for family planning, C denote the number of modern contraceptive users, T denote the number of modern and traditional contraceptive users, U denote the number with unmet need for family planning. Then we have mCPR = C/P, % unmet need = U/P, and % met need (or satisfied demand) = C/N.

$$\%\, satisfied\ demand = \frac{C}{N} = \frac{C}{T+U} = \frac{mCPR}{CPR + \%unmet\ need}$$

An increase in C implies higher mCPR, but it does not necessarily increase % satisfied demand. The relationship between the indicators becomes complex in other scenarios, such as when more women express a need for family planning. This will decrease the % met need without affecting mCPR. The congruence, and lack of it, has been observed in FP2020 countries. From 2012 to 2017, the high growth of mCPR has driven a nine-percentage point increase in demand satisfied in Eastern and Southern Africa. During the same period, Central and West Africa experienced comparable mCPR growth, but that was accompanied by increasing levels of unmet need. These

For peer review only - http://bmjopen.bmj.com/site/about/guidelines.xhtml

are the results of a complex dynamic involving both fertility intentions and available family planning services. As a result, our subsequent empirical analyses will be based on probabilistic statistical regression rather than deterministic mathematical relationships.

Another complicating factor is that FP2020 counts all women, irrespective of their marital status, while the 75% target only covers married or in-union women. Although subsequent debates consider expanding the satisfied demand target to all women, no consensus has been reached, and therefore we will use the original statement of the 75% target. The difference in denominators will be dealt with in our statistical models.

The congruence between FP2020 and 75% satisfied demand targets requires a bi-directional assessment. We estimated the implications of achieving one of them on the other. Specifically, the study attempts to answer the following two questions: (1) how many additional users will be added following the 75% satisfied demand target; (2): what percentage of demand will be satisfied in 41 commitment-making countries assuming an annual increase of 1.4 percentage points from 2012 until 2030? Annual growth of 1.4% is the overall target proposed by the London Summit on Family Planning Metrics Group across all FP2020 focus countries [9]. Overall annual growth of 0.7 percentage points was observed across the world's 69 poorest countries before 2012. Brown et al. estimated that doubling the annual growth to 1.4 would add 120 million modern contraceptive users by 2020. The target growth rate is considered an aspirational yet achievable goal assuming the resources and leadership around current family planning programs may be collectively mobilized. These two assessments are conducted separately, albeit employing a similar methodology (Figure 1).

For peer review only - http://bmjopen.bmj.com/site/about/guidelines.xhtml

<Figure 1 about here>

There are three steps to answer the first research question. The first step is to estimate the necessary married-woman mCPR to satisfy 75% demand with modern methods by 2030. Among the 41 commitment-making countries, five FP2020 commitment-making countries had already reached the 75% satisfied demand goal in their most recent surveys (Table 1). It is reasonable to assume that maintaining at least 75% satisfied demand by 2030 is the goal in those countries. We assume the mCPR and % satisfied demand will remain at their most recent observed level until 2030.

<Table 1 about here>

For the other 36 countries, the percentage of demand satisfied with modern methods is assumed to reach 75% in 2030. Then we employ the following country-level fixed effects longitudinal model to estimate the required mCPR for the assumed 75% satisfied demand.

$$y_{it} = \beta_0 + \beta_1 x_{it} + \beta_2 x_{it}^2 + \alpha_i + \varepsilon_{it} \quad (1)$$

where y_{it} denote the mCPR for country i in time t; x_{it} denotes the % satisfied demand for country i in time t; α_i denotes the time-invariant unobserved fixed effects for country i; ε_{it} denotes the error term. The mode is chosen from several options due to its best predictive performance. The model is first fitted using survey-based data compiled by the United Nations.

For peer review only - http://bmjopen.bmj.com/site/about/guidelines.xhtml

The Least Squares Dummy Variable (LSDV) method is used in the model estimation [15]. This approach explicitly provides the coefficients of the country dummy, which is required in predicting the mCPR for the assumed 75% satisfied demand in 2030. Then with the estimated coefficients and country-level fixed effects, we estimate mCPR for the assumed 75% satisfied demand.

The second step is to convert the married-woman mCPR estimated in step 1 to all-woman mCPR. Two hundred sixty-two DHS surveys based on samples of all women of reproductive ages were conducted from 1990 to 2016 in 85 countries. We use the following fixed effects longitudinal model to estimate all-woman mCPR from married mCPR

$$a_i = \theta_0 + \theta_1 m_i + v_i + \epsilon_{it} \quad (2)$$

where a_i and m_i denote the all-woman and married mCPR in survey i; v_i denotes region (SSA vs. non-SSA) level fixed effects. We use region level instead of country-level fixed effects because a model with country-level fixed effects cannot be used for prediction in FP2020 countries without a DHS survey.

In the third step, we assume all-woman mCPR will increase linearly from the level in the last survey to the level estimated for 2030 in step 2. Using the number of women of reproductive age obtained from World Population Prospects 2017, we calculate the number of modern contraceptive users in the 67 FP2020 focus countries.[16]

For peer review only - http://bmjopen.bmj.com/site/about/guidelines.xhtml

The second research question is answered similarly in three steps (Figure 1). We first estimate the baseline, i.e., all-woman mCPR in 2012. Our principle is to rely on the survey-based estimates as much as possible. As mentioned above, 5 of the 41 FP2020 commitment-making countries had already reached the 75% satisfied demand goal in their most recent surveys, and therefore are excluded from this investigation. Among the other 36 commitment-making countries, 10 conducted a survey in 2012. For those 19 countries that have conducted surveys both before and after 2012, we use the two surveys before and after 2012 to linearly interpolate the mCPR for 2012. For the other 7 countries that only have surveys conducted before 2012, we used the last survey-based estimate for 2012.

Then we impose a 1.4% annual increase in all-woman mCPR from 2012 until 2030. Finally, we predict the % satisfied demand associated with the calculated levels of all-woman mCPR for 2012-2030 based on a fixed effects longitudinal model similar to Equation (1), but moving % satisfied demand to the left-hand side and including mCPR and its squared term in the right-hand side.

Patient and Public Involvement

The study does not involve patients or the public.

Results

All three fixed effects longitudinal models fit the data quite well, indicating excellent predictive performance (Table 2). Using 466 survey-based estimates, the adjusted R-squared of the model regressing married-woman mCPR on % satisfied demand and a country dummy is above 0.98,

For peer review only - http://bmjopen.bmj.com/site/about/guidelines.xhtml

meaning that less than 2% of the variations in married-woman mCPR cannot be explained by the model (Model 1). As a result, the estimated married-woman mCPR based on the assumed 75% satisfied demand should be highly accurate and reliable. The adjusted R-squared of 0.97 in Model 2 also indicates accurate conversion from married- to all-woman mCPR. Model 3 that regresses % satisfied demand on married-woman mCPR also performed well (adjusted R-squared 0.97).

<Table 2 about here>

Achieving the 75% satisfied demand by 2030 goal means a gain of approximately 82 million additional users in these 67 FP2020 countries from 2012 to 2020, which is about 68% of the 120 million proposed by the FP2020 Initiative (Table 3). From 2012 to 2020, these 41 commitment-making countries will contribute 74 million additional users while these 26 non-commitment-making FP2020 countries contribute 8 million. If the 67 countries continue the mCPR growth rate implied by the 75% satisfied demand initiative, the goal of adding 120 million modern contraceptive users will be achieved in early 2023 (Figure 2). By 2030, there will be 184 and 21 million additional users in commitment-making and non-commitment-making countries, respectively, making a total number of 206 additional modern contraceptive users in these 67 FP2020 countries.

<Table 3 about here>

For peer review only

Five of the 41 FP2020 commitment-making countries (three in sub-Saharan Africa)have already satisfied 75% or more of the contraceptive demand, according to their last survey. Among the other 36 commitment-making countries, only four additional countries (Bangladesh, India, Malawi, and Vietnam) will reach that target by 2020, following FP2020's proposed 1.4% annual increase in mCPR (Table 4). Another eight countries (Ethiopia, Laos, Madagascar, Nepal, Rwanda, Solomon Islands, Tanzania, and Zambia) will do so by 2030. Disaggregated by region, the situation is more challenging in sub-Saharan Africa, where only one (Malawi) of the 26 commitment-making countries will reach the 75% target by 2020, and another five countries (Ethiopia, Madagascar, Rwanda, Tanzania, and Zambia) will do so by 2030. Adding those three countries that had already reached the target in their most recent surveys, less than one third (9) of the 29 commitment-making countries in this region will satisfy 75% demand for family planning by 2030. In the other regions, five countries will achieve the target by 2020, and another three will do so by 2030. Those eight target-achieving countries represent two-thirds of the 12 non-SSA commitment-making countries.

In sum, assuming FP2020's proposed annual growth rate in mCPR, the % satisfied will reach 75% in less than half (17) of the 41 FP2020 commitment-making countries

<Table 4 about here>

Discussion

The contribution of this study is an improved understanding of the convergence of targets of two global family planning initiatives: FP2020's adding 120 modern contraceptive users by 2020 in

For peer review only - http://bmjopen.bmj.com/site/about/guidelines.xhtml

69 of the world's poorest countries and USAID's satisfying 75% demand for family planning with modern contraceptives. We estimate and discuss the implication of reaching one target on the other. The shared goal may facilitate building a broad coalition to promote family planning in the developing world. These two initiatives represent the objectives of two major donors to family planning.[17] The % satisfied demand has also been adopted as an indicator in the Sustainable Development Goals. Due to their different features and advantages, mCPR (and number of users) and % satisfied demand will continue coexisting in the international agenda for family planning. Despite their theoretical correlation, the empirical relation between the two indicators depends on other context-specific factors, such as demand generation and changes in fertility desire. As a result, an assessment of the empirical correlation between the two indicators has sustaining policy implications. A consensus goal is critical to building a broad coalition to collectively and effectively mobilize financial and political resources and capture global attention.

Our results show that the two initiatives move towards the same goal of promoting access to family planning for women and girls. Overall, both the 75% satisfied demand and the FP2020 goal are ambitious. Achieving the 75% satisfied demand goal by 2030 implies that 82 million or 68% of the 120-million target users will be added by 2020 in 67 FP2020 focus countries. The target of 120 million will be achieved by 2023, only three years later than the FP2020 deadline. On the other hand, achieving a 1.4% annual increase in all-woman mCPR will enable only 17 of the 41 commitment-making countries to attain the goal of 75% satisfied demand by 2030. The overall assessment should not mask the across-country variations. In some countries it is more plausible to achieve the FP2020's proposed annual increase of 1.4 percentage points than

For peer review only - http://bmjopen.bmj.com/site/about/guidelines.xhtml

satisfying the 75% demand by 2030. Some other countries, however, have satisfied 75% demand or will do so by 2030 with an annual mCPR increase below 1.4 percentage points.

The simulated implications of achieving one target on the other have several policy implications, which are urgently needed as donors and stakeholders are debating about the post-FP2020 plan. First, multiple measures will continue coexisting in international family planning. The FP2020 Core Group of which the Bill and Melinda Gates Foundation, United Kingdom's Department for International Development (DFID), USAID and United Nations Population Fund (UNFPA) are active may renew their commitment to adding modern contraceptive users beyond FP2020 to the FP2030 deadline. The % satisfied demand has been adopted as an indicator as SDG 3.7.1. The 75% benchmark is being used as a proxy for the minimum definition of "universal access to reproductive health" in terms of contraceptive use (SDG 3). Methodologically, our models for assessing the congruence of the two measures could be replicated as the FP2020 movement sets its goals for FP2030.

Second, our exercise sheds light on the choice between aspirational and realistic target-setting approaches. The findings show that 75% satisfied demand can be viewed in three settings: 1) countries who have already achieved the goal but whose plans involve increasing the percentage higher than the 75% benchmark (e.g., Indonesia, Myanmar, Kenya, South Africa), Zimbabwe); 2) countries which are projected to likely reach the goal by 2020 and 2030; and 3) countries which will remain below the goal (21 of 41 commitment-making countries). With only one year left before its deadline, FP2020 has contributed to the mobilization of global resources for family

For peer review only - http://bmjopen.bmj.com/site/about/guidelines.xhtml

planning and has shown progress against the goal but not at the trajectory to reach 120 million more women and girls by 2020.

The third policy implication is for the choice between global and national targets. All countries in our exercise belong to low-income countries, but they still demonstrate massive diversity in terms of mCPR, desired and realized fertility, and population age structure. When setting targets in the future, donors and stakeholders need to strike a balance between simplification (global target as in FP2020) and customization (country-specific targets as in 75% satisfied demand).

The last policy implication is on SDG. Although % satisfied demand has been adopted as an indicator (SDG 3.7.1), it has not been associated with quantitative goals. The same situation occurred to Target 5b of Millennium Development Goals (MDGs): "Achieve, by 2015, universal access to reproductive health". Several studies argued that clear, measurable goals can be a focal point for coalescing political support for action.[9] Reflecting on the lag in substantively integrating family planning into the MDGs, FP2020 proposed a quantifiable target of adding 120 million modern contraceptive users by 2020. Adopting the target of 75% satisfied demand in SDG may help mobilize and guide resource allocation and provide a benchmark for program advocacy. Per our simulation results, the target is achievable in certain countries and aspirational in others.

The study is not without limitations. First, despite the highly satisfactory model fit, our regressions could be theoretically improved by including other factors such as calendar time. We did not include year as a covariate because its coefficient reflects not only temporal effects but

For peer review only - http://bmjopen.bmj.com/site/about/guidelines.xhtml

also the changing composition of countries in the database. For example, the earliest DHS surveys were mostly in Africa, while Asia was added later. So, the absence of calendar time in the model is a limitation with the database rather than our methodology. Since we are mainly interested in the predictive performance of the model, measured by the adjusted R-squared, and adding year as a covariate changed the adjusted R-squared by less than 1 percentage point, our final model did not consider calendar time. The second limitation is the linear assumption on mCPR growth. Other growth curves (such as S-shaped or logistic) may be more accurate in many countries. The 67 FP2020 countries are in different stages of mCPR growth, some experiencing a convex trajectory and some a concave trajectory. Fully accounting for country-specific curves will likely make the statistical models much more complex and less robust. We believe a linear trajectory provides an acceptable approximation for the mixture of convex and concave trajectories. Consequently, the global estimates presented in the study may not be substantially affected by the assumed linearity.

As repeatedly emphasized in the London Summit document, setting a quantitative target should not cause concern among those firmly committed to sexual and reproductive health and rights because all interventions will have women's rights at the center of their implementation efforts. Our assessment in this study of the congruence of major, articulated family planning initiatives aims to unite international communities into collective actions that secure women's and girls' access to effective contraceptive methods.

Author contributions: QL, SA, and JR devised the study and wrote the article. QL compiled the data and led the statistical modeling and analysis.

For peer review only - http://bmjopen.bmj.com/site/about/guidelines.xhtml

Disclosure statement: No potential conflict of interest was reported by the authors.

Ethics and consent: This study only uses publicly available secondary data and does not involve any participants. Therefore, ethical approval and participant consent are not required.

Funding: Bill and Melinda Gates Foundation

References

1. Cleland J, Bernstein S, Ezeh A, Faundes A, Glasier A, Innis J. Family planning: the unfinished agenda. *The Lancet* 2006; **368**(9549): 1810-27.
2. Ahmed S, Li Q, Liu L, Tsui AO. Maternal deaths averted by contraceptive use: an analysis of 172 countries. *The Lancet* 2012; **380**(9837): 111-25.
3. Cleland J, Conde-Agudelo A, Peterson H, Ross J, Tsui A. Contraception and health. *Lancet (London, England)* 2012; **380**(9837): 149-56.
4. Canning D, Schultz TP. The economic consequences of reproductive health and family planning. *Lancet (London, England)* 2012; **380**(9837): 165-71.
5. Cohen SA. London summit puts family planning back on the agenda, offers new lease on life for millions of women and girls. *Guttmacher Policy Review* 2012; **15**(3): 20-4.
6. Ezeh AC, Bongaarts J, Mberu B. Global population trends and policy options. *Lancet (London, England)* 2012; **380**(9837): 142-8.
7. Shiffman J, Quissell K. Family planning: a political issue. *Lancet (London, England)* 2012; **380**(9837): 181-5.
8. Kim JY, Ammann A. Is the "3 by 5" Initiative the Best Approach to Tackling the HIV Pandemic? *PLoS Medicine* 2004; **1**(2): e37.
9. Brown W, Druce N, Bunting J, et al. Developing the "120 by 20" goal for the Global FP2020 Initiative. *Stud Fam Plann* 2014; **45**(1): 73-84.
10. Fabic MS, Choi Y, Bongaarts J, et al. Meeting demand for family planning within a generation: the post-2015 agenda. *Lancet (London, England)* 2015; **385**(9981): 1928-31.
11. Choi Y, Short Fabic M. Monitoring Progress in Equality for the Sustainable Development Goals: A Case Study of Meeting Demand for Family Planning. *Global health, science and practice* 2018; **6**(2): 390-401.
12. United Nations General Assembly. Sustainable Development Goals 3. https://sustainabledevelopment.un.org/sdg3 (accessed August 9 2019).
13. Cahill N, Sonneveldt E, Stover J, et al. Modern contraceptive use, unmet need, and demand satisfied among women of reproductive age who are married or in a union in the focus countries of the Family Planning 2020 initiative: a systematic analysis using the Family Planning Estimation Tool. *Lancet (London, England)* 2017.
14. United Nations DoEaSA, Population Division (2017). World Contraceptive Use 2017 (POP/DB/CP/Rev2017).
15. Greene WH. Econometric analysis: Prentice Hall; 2011.
16. United Nations DoEaSA, Population Division, W. World Population Prospects: The 2017 Revision, Key Findings and Advance Tables. Working Paper No. ESA/P/WP/248. 2017.
17. IHME. Financing Global Health 2014: Shifts in Funding as the MDG Era Closes. Seattle, WA: IHME; 2015.

For peer review only - http://bmjopen.bmj.com/site/about/guidelines.xhtml

Figure 1: Analytical flowchart for the two research questions

Figure 2: Number of additional modern contraceptive users in 41 commitment-making and 26 non-commitment-making countries assuming the trajectory of satisfying 75% demand by 2030

For peer review only

For peer review only - http://bmjopen.bmj.com/site/about/guidelines.xhtml

Table 1: Modern contraceptive prevalence rate (mCPR) in 5 commitment-making countries where % demand satisfied exceeded 75% in the last survey

Country	Region	Survey date	mCPR	% demand satisfied
Myanmar	Non-SSA	2015-16	51.3	75.0
Kenya	SSA	2015	62.6	76.2
Indonesia	Non-SSA	2015-16	59.5	78.8
South Africa	SSA	2003-04	59.8	81.1
Zimbabwe	SSA	2015	65.8	85.2

Notes: SSA denotes sub-Sahara Africa; non-SSA includes all other regions

For peer review only

For peer review only - http://bmjopen.bmj.com/site/about/guidelines.xhtml

Table 2: Goodness of fit of the fixed effects longitudinal models

	Model 1	Model 2	Model 3
**Outcome**	Married mCPR	All-woman mCPR	% satisfied demand
**Covariates**	% satisfied demand	Married mCPR	Married mCPR
	(% satisfied demand)^2		(married mCPR)^2
**Fixed effects**	Country level	Region (SSA; non-SSA) level	Country level
**Sample size**	466	262	466
**R-squared**	0.98	0.91	0.97

For peer review only

For peer review only - http://bmjopen.bmj.com/site/about/guidelines.xhtml

Table 3: Modern contraceptive prevalence rate (mCPR), modern contraceptive users (thousand), and added users since 2012 (thousand) in 41 commitment-making and 26 non-commitment-making countries under FP2020: assuming the achievement of 75% satisfied demand by 2030

Country	2012		2020			2030		
	mCPR	Users	mCPR	Users	Added users	mCPR	Users	Added users
**Commitment-making countries (41)**								
Afghanistan	10.4	691	22.7	2,065	1,374	38.1	4,645	3,954
Bangladesh	41.2	17,800	42.4	20,200	2,366	43.8	22,200	4,369
Benin	7.0	158	22.0	636	478	40.9	1,570	1,412
Burkina Faso	13.5	515	25.5	1,249	734	40.5	2,704	2,189
Burundi	16.7	371	27.5	768	397	41.0	1,599	1,229
Cameroon	14.2	708	25.8	1,621	913	40.3	3,362	2,655
Chad	0	0	17.2	651	651	39.3	2,052	2,052
Côte d'Ivoire	11.5	566	24.2	1,509	943	39.9	3,277	2,711
DR Congo	3.9	594	20.3	4,066	3,472	40.8	11,600	11,000
Ethiopia	26.2	5,677	32.6	9,285	3,608	40.5	15,100	9,398
Ghana	14.3	935	26.9	2,093	1,158	42.6	4,080	3,145
Guinea	4.8	124	20.1	655	531	39.2	1,711	1,588
Haiti	24.5	663	32.9	1,009	346	43.3	1,493	829
India	35.7	115,000	38.5	138,000	22,600	41.9	162,000	46,700
Indonesia	45.7	31,000	45.7	32,900	1,904	45.7	34,600	3,521
Kenya	47.1	5,076	47.7	6,592	1,516	48.5	8,575	3,499
Laos	33.4	570	38.1	742	171	44.1	972	401
Liberia	14.2	139	25.9	318	179	40.6	649	510
Madagascar	26.2	1,395	32.8	2,246	851	41.0	3,674	2,280
Malawi	45.4	1,696	44.7	2,218	522	44.0	2,984	1,289
Mali	7.6	268	21.2	962	694	38.1	2,427	2,160
Mauritania	7.6	70	22.2	259	189	40.5	610	540

For peer review only

For peer review only - http://bmjopen.bmj.com/site/about/guidelines.xhtml

Mozambique	11.3	676	23.3	1,792	1,115	38.2	4,002	3,325
Myanmar	39.5	5,503	39.5	5,956	453	39.5	6,197	694
Nepal	34.0	2,504	38.1	3,293	789	43.4	4,078	1,574
Niger	5.2	192	19.2	971	778	36.6	2,781	2,588
Nigeria	6.2	2,364	20.5	9,773	7,409	38.5	24,200	21,900
Pakistan	20.0	8,935	29.5	15,400	6,475	41.3	26,100	17,100
Philippines	28.1	7,022	35.8	10,100	3,100	45.5	14,700	7,664
Rwanda	36.1	974	39.2	1,326	353	43.1	1,880	906
Senegal	12.8	429	24.7	1,040	611	39.6	2,217	1,787
Sierra Leone	11.6	188	23.7	479	291	38.8	997	809
Solomon Islands	24.1	33	28.0	45	13	32.7	64	31
South Africa	46.4	6,715	46.4	7,366	651	46.4	8,067	1,352
South Sudan	6.3	160	21.2	697	537	39.9	1,715	1,555
Togo	11.8	195	25.1	516	322	41.7	1,109	915
Uganda	20.5	1,652	30.7	3,355	1,703	43.4	6,715	5,062
Tanzania	21.8	2,483	30.1	4,459	1,976	40.4	8,243	5,760
Viet Nam	43.4	11,200	45.8	11,900	686	48.8	12,600	1,401
Zambia	34.3	1,176	38.0	1,731	555	42.7	2,627	1,451
Zimbabwe	50.9	1,944	50.9	2,366	422	50.9	3,010	1,066
**Subtotal**		**238,350**		**312,597**	**73,836**		**423,176**	**184,372**
**Non-commitment-making countries (26)**								
Bhutan	50.2	101	50.2	114	14	50.2	124	23
Bolivia	30.5	788	37.6	1,129	341	46.6	1,590	801
Cambodia	28.5	1,159	35.1	1,581	422	43.3	2,281	1,121
Central African Republic	12.7	133	24.7	292	158	39.7	627	493
Comoros	12.0	21	25.3	54	33	41.9	114	92
Congo	10.9	120	24.9	334	214	42.3	755	635

For peer review only - http://bmjopen.bmj.com/site/about/guidelines.xhtml

Egypt	43.8	9,961	43.8	11,200	1,256	43.8	13,200	3,262
Eritrea	9.9	108	23.1	311	203	39.6	704	595
Gambia	5.6	24	19.8	109	85	37.6	282	258
Guinea-Bissau	10.2	41	18.1	90	49	27.9	180	139
Honduras	49.0	1,104	49.0	1,319	215	49.0	1,495	391
Iraq	28.5	2,270	33.5	3,367	1,097	39.6	5,157	2,887
Kyrgyzstan	26.3	401	32.1	501	100	39.4	703	301
Lesotho	46.4	251	46.4	290	39	46.4	337	86
Mongolia	36.9	305	39.5	331	26	42.8	392	87
Nicaragua	59.2	960	59.2	1,053	93	59.2	1,119	159
North Korea	58.5	3,899	58.5	3,800	-100	58.5	3,654	-246
Palestine	33.1	348	37.0	490	143	41.9	717	369
Papua New Guinea	24.6	449	32.5	717	267	42.3	1,130	680
Sao Tome and Principe	27.3	12	36.2	19	7	47.4	32	20
Sri Lanka	42.1	2,251	44.6	2,374	123	47.6	2,473	222
Sudan	6.5	552	21.1	2,240	1,688	39.3	5,425	4,873
Tajikistan	20.3	428	28.0	670	242	37.5	1,086	657
Timor-Leste	18.2	46	24.0	74	28	31.1	127	81
Uzbekistan	39.9	3,334	40.1	3,597	263	40.3	3,964	630
Yemen	21.8	1,326	30.3	2,339	1,013	41.0	4,079	2,753
**Subtotal**		**30,395**		**38,397**	**8,019**		**51,744**	**21,372**
**Total (67 focus countries)**		**268,745**		**350,995**	**81,855**		**474,920**	**205,744**

Notes: the predicted mCPR for Chad 2012 was rounded to 0; the columns may add up exactly because our statistical models used exact numbers while results are presented in thousands.

For peer review only - http://bmjopen.bmj.com/site/about/guidelines.xhtml

Table 4: Modern contraceptive prevalence rate (mCPR) and % demand satisfied (%SD) in 2012, 2020 and 2030 in 36 commitment-making countries: assuming the achievement of FP2020 and extending its mCPR trajectories to 2030

Country	2020		2030	
	mCPR	%SD	mCPR	%SD
**Sub-Sahara Africa(26)**				
Benin	19.1	33.7	33.1	52.0
Burkina Faso	27.4	47.1	41.4	63.5
Burundi	28.9	46.5	42.9	62.5
Cameroon	27.8	48.3	41.8	64.5
Chad	15.9	31.4	29.9	50.5
Côte d'Ivoire	23.7	42.6	37.7	59.8
DR Congo	18.8	33.5	32.8	51.9
Ethiopia	40.9	63.6	54.9	**76.7**
Ghana	32.8	50.5	46.8	65.6
Guinea	15.8	31.8	29.8	50.9
Liberia	28.9	49.0	42.9	65.0
Madagascar	40.4	61.4	54.4	**74.7**
Malawi	62.1	**77.7**	76.1	**85.8**
Mali	21.1	42.6	35.1	60.5
Mauritania	22.7	40.8	36.7	58.3
Mozambique	22.5	45.6	36.5	63.1
Niger	23.4	49.9	37.4	67.2
Nigeria	22.0	44.7	36.0	62.3
Rwanda	56.8	73.3	70.8	**82.6**
Senegal	27.3	48.6	41.3	65.0
Sierra Leone	24.9	47.9	38.9	64.9
South Sudan	12.9	23.8	26.9	43.6
Togo	26.9	42.8	40.9	59.3
Uganda	37.0	54.6	51.0	68.7
Tanzania	40.5	63.8	54.5	**77.0**
Zambia	53.1	72.0	67.1	**82.2**
**Other regions (10)**				
Afghanistan	31.0	56.9	45.0	72.4
Bangladesh	70.5	**83.1**	84.5	**89.2**
Haiti	42.5	59.7	56.5	72.5
India	59.1	**78.0**	73.1	**86.8**
Laos	53.9	70.1	67.9	**80.2**

For peer review only - http://bmjopen.bmj.com/site/about/guidelines.xhtml

Nepal	54.4	72.1	68.4	**82.0**
Pakistan	37.3	57.7	51.3	71.6
Philippines	48.3	62.7	62.3	74.0
Solomon Islands	38.6	73.3	52.6	**87.0**
Viet Nam	69.7	**77.3**	83.7	**83.6**

Notes: bold indicates reaching the target of satisfying 75% demand for family planning; Madagascar's 74.7% in 2030 can be rounded to 75%.

For peer review only

For peer review only - http://bmjopen.bmj.com/site/about/guidelines.xhtml

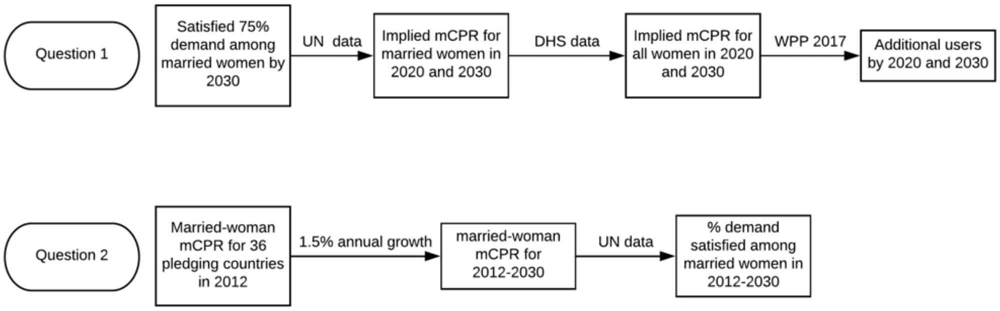

Figure 1: Analytical flowchart for the two research questions

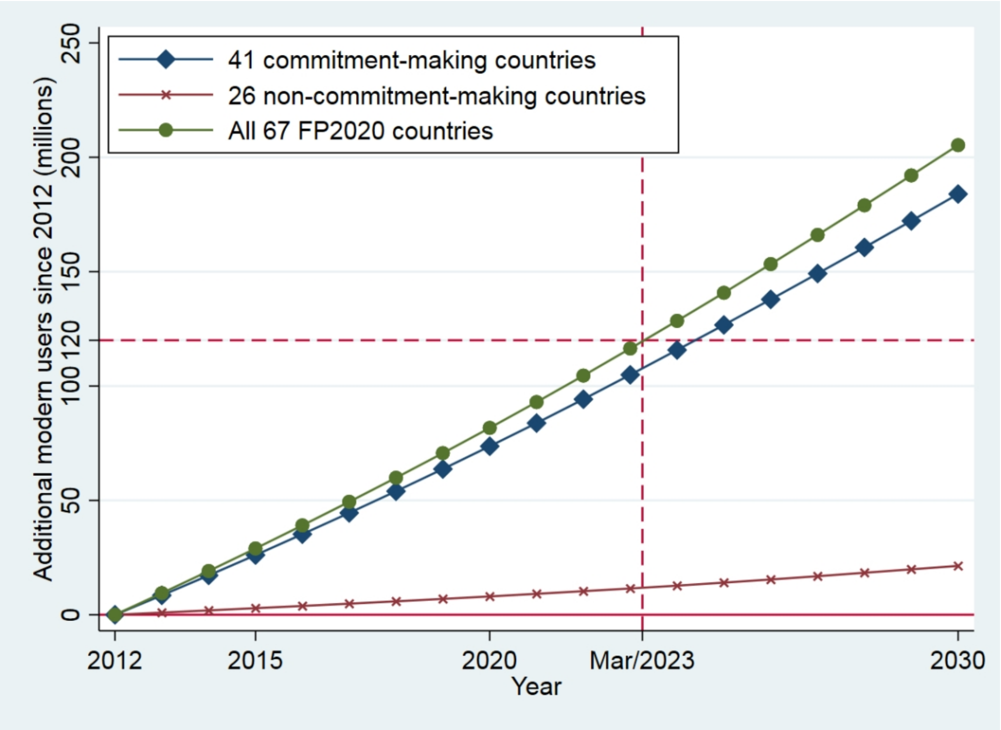

Figure 2: Number of additional modern contraceptive users in 41 pledging and 26 non-pledging countries assuming the trajectory of satisfying 75% demand by 2030

423x308mm (72 x 72 DPI)

STROBE Statement—checklist of items that should be included in reports of observational studies

	Item No.	Recommendation	Page No.
**Title and abstract**	1	(*a*) Indicate the study's design with a commonly used term in the title or the abstract	1
		(*b*) Provide in the abstract an informative and balanced summary of what was done and what was found	2-3
**Introduction**			
Background/rationale	2	Explain the scientific background and rationale for the investigation being reported	4-6
Objectives	3	State specific objectives, including any prespecified hypotheses	6
**Methods**			
Study design	4	Present key elements of study design early in the paper	6
Setting	5	Describe the setting, locations, and relevant dates, including periods of recruitment, exposure, follow-up, and data collection	6-7
Participants	6	(*a*) *Cohort study*—Give the eligibility criteria, and the sources and methods of selection of participants. Describe methods of follow-up<br>*Case-control study*—Give the eligibility criteria, and the sources and methods of case ascertainment and control selection. Give the rationale for the choice of cases and controls<br>*Cross-sectional study*—Give the eligibility criteria, and the sources and methods of selection of participants	
		(*b*) *Cohort study*—For matched studies, give matching criteria and number of exposed and unexposed<br>*Case-control study*—For matched studies, give matching criteria and the number of controls per case	
Variables	7	Clearly define all outcomes, exposures, predictors, potential confounders, and effect modifiers. Give diagnostic criteria, if applicable	6-8
Data sources/ measurement	8*	For each variable of interest, give sources of data and details of methods of assessment (measurement). Describe comparability of assessment methods if there is more than one group	*6-11*
Bias	9	Describe any efforts to address potential sources of bias	6-11
Study size	10	Explain how the study size was arrived at	6

Continued on next page

For peer review only - http://bmjopen.bmj.com/site/about/guidelines.xhtml
For peer review only

Quantitative variables	11	Explain how quantitative variables were handled in the analyses. If applicable, describe which groupings were chosen and why	6-8
Statistical methods	12	(*a*) Describe all statistical methods, including those used to control for confounding	6-11
		(*b*) Describe any methods used to examine subgroups and interactions	
		(*c*) Explain how missing data were addressed	
		(*d*) *Cohort study*—If applicable, explain how loss to follow-up was addressed<br>*Case-control study*—If applicable, explain how matching of cases and controls was addressed<br>*Cross-sectional study*—If applicable, describe analytical methods taking account of sampling strategy	
		(*e*) Describe any sensitivity analyses	
**Results**			
Participants	13*	(a) Report numbers of individuals at each stage of study—eg numbers potentially eligible, examined for eligibility, confirmed eligible, included in the study, completing follow-up, and analysed	
		(b) Give reasons for non-participation at each stage	
		(c) Consider use of a flow diagram	
Descriptive data	14*	(a) Give characteristics of study participants (eg demographic, clinical, social) and information on exposures and potential confounders	
		(b) Indicate number of participants with missing data for each variable of interest	
		(c) *Cohort study*—Summarise follow-up time (eg, average and total amount)	
Outcome data	15*	*Cohort study*—Report numbers of outcome events or summary measures over time	
		Case-control study—Report numbers in each exposure category, or summary measures of exposure	
		Cross-sectional study—Report numbers of outcome events or summary measures	
Main results	16	(*a*) Give unadjusted estimates and, if applicable, confounder-adjusted estimates and their precision (eg, 95% confidence interval). Make clear which confounders were adjusted for and why they were included	11-13
		(*b*) Report category boundaries when continuous variables were categorized	
		(*c*) If relevant, consider translating estimates of relative risk into absolute risk for a meaningful time period	

Continued on next page

For peer review only

Other analyses	17	Report other analyses done—eg analyses of subgroups and interactions, and sensitivity analyses	
**Discussion**			
Key results	18	Summarise key results with reference to study objectives	11-13
Limitations	19	Discuss limitations of the study, taking into account sources of potential bias or imprecision. Discuss both direction and magnitude of any potential bias	16-17
Interpretation	20	Give a cautious overall interpretation of results considering objectives, limitations, multiplicity of analyses, results from similar studies, and other relevant evidence	17
Generalisability	21	Discuss the generalisability (external validity) of the study results	17
**Other information**			
Funding	22	Give the source of funding and the role of the funders for the present study and, if applicable, for the original study on which the present article is based	18

*Give information separately for cases and controls in case-control studies and, if applicable, for exposed and unexposed groups in cohort and cross-sectional studies.

Note: An Explanation and Elaboration article discusses each checklist item and gives methodological background and published examples of transparent reporting. The STROBE checklist is best used in conjunction with this article (freely available on the Web sites of PLoS Medicine at http://www.plosmedicine.org/, Annals of Internal Medicine at http://www.annals.org/, and Epidemiology at http://www.epidem.com/). Information on the STROBE Initiative is available at www.strobe-statement.org.

For peer review only

Capitalizing on shared goals for family planning: a concordance assessment of two global initiatives using longitudinal statistical models

Journal:	*BMJ Open*
Manuscript ID	bmjopen-2019-031425.R2
Article Type:	Original research
Date Submitted by the Author:	08-Oct-2019
Complete List of Authors:	Li, Qingfeng; Johns Hopkins Bloomberg School of Public Health, International Health<br>Rimon, Jose; Johns Hopkins Bloomberg School of Public Health, Population, Family and Reproductive Health<br>Ahmed, Saifuddin; Johns Hopkins University, Population, family and reproductive Health; Johns Hopkins University
**Primary Subject Heading**:	Global health
Secondary Subject Heading:	Public health
Keywords:	Family planning, Global initiatives, FP2020, 75% satisfied demand

SCHOLARONE™
Manuscripts

Capitalizing on shared goals for family planning: a concordance assessment of two global initiatives using longitudinal statistical models

Qingfeng Li[1,2], Jose G. Rimon[2], Saifuddin Ahmed[2]

[1]Department of International Health
[2]Bill & Melinda Gates Institute for Population and Reproductive Health
Department of Population, Family and Reproductive Health
Johns Hopkins Bloomberg School of Public Health, Baltimore, Maryland, United States of America

Corresponding author:
Qingfeng Li, MHS, PhD
Bloomberg School of Public Health
Johns Hopkins University
615 North Wolfe Street E8642
Baltimore, MD 21205
Tel:(410) 614-4890
e-mail: qli28@jhu.edu

Keywords: Family planning, Global initiatives, FP2020, 75% satisfied demand

Word Count: Abstract: 291; Body: 3555

For peer review only - http://bmjopen.bmj.com/site/about/guidelines.xhtml

Objective: Family planning is unique among health interventions in its breadth of health, development and economic benefits. The complexity of formulating effective strategies to promote women's and girls' access to family planning calls for closer coordination of resources and attention from all stakeholders. Our objective was to quantify the concordance of two global initiatives: Family Planning 2020's adding 120 million modern contraceptive users by 2020 (proposed during The London Summit 2012 by Gates Foundation) and satisfying the 75% demand for modern contraceptives by 2030 (proposed by United States Agency for International Development). A demonstration of their concordance, or lack thereof, provides an understanding of the proposed quantitative goals and helps to formulate collective strategies.

Design and setting: We applied fixed effects longitudinal models to assess the convergence of the two initiatives. The implications of success in one initiative on achieving the other are simulated to illustrate their shared goals. Publicly available data on contraceptive use, unmet need, and met need from national surveys are used. Extensive model validations were conducted to check and confirm models' predictive performance.

Results: Our results show that the 75% satisfied demand initiative will reach 82 million additional modern users by 2020 and 120 million by early 2023. Following FP2020's proposed annual increase of modern contraceptive use, nine of the 41 commitment-making countries will reach the 75% target by 2020; another eight countries will do so by 2030. Extending FP2020's proposed contraceptive growth to 2030 implies the achievement of the 75% target in less than half (17) of the 41 commitment-making countries.

For peer review only

Conclusions: The results from the statistical exercise demonstrate that the two global initiatives move toward the same goal of promoting access to FP and overall both are ambitious. Closer coordination between major stakeholders in international family planning may stimulate more efficient mobilization and utilization of global sources, which is urgently needed to accelerate the progress toward satisfying women's need for family planning.

Strengths of this study

- This study is the first systematic comparison of two major global initiatives on family planning
- The estimations are based on rigorously developed and validated statistical models
- The findings provide new insights into the shared goals of the two initiatives and have important policy implications

Limitations of this study

- Relying on secondary data restricts variable selection for the statistical models.
- The linear growth curve assumed for modern contraceptive prevalence rate (mCPR) in the study may not be accurate for each country.

For peer review only

For peer review only - http://bmjopen.bmj.com/site/about/guidelines.xhtml

Introduction

Access to family planning is a critical component of reproductive rights and leads to multi-faceted benefits for women and their families. It is unique among health interventions in its breadth of health, development and economic benefits, such as reducing maternal and child mortality, empowering women and girls, and enhancing environmental sustainability.[1,2] The Lancet series on family planning in 2012 documented strong evidence of the extensive gains resulting from family planning. Ahmed and colleagues estimated that contraceptive use in 172 countries averted 272,040 maternal deaths in 2008, and satisfying unmet need for contraceptive methods could prevent another 104,000 deaths per year.[2] Cleland and colleagues made nearly identical estimates using a different methodology.[3] Additionally, Canning and Schultz evaluated the economic consequences of family planning, including increases in female labor force participation and proportion in paid employment.[4]

However, after reaching their global peak following the 1994 International Conference on Population and Development (ICPD) in Cairo, both financial support and political commitments for family planning have plateaued, and even declined in many countries, in the decade prior to 2012.[1,5] Consequently, progress towards providing access to contraception for women and girls in developing countries has been slow. On average, women in Sub-Saharan Africa continue to have more than five children.[6]

Compared with other public health interventions, family planning has two unique features that need special attention. First, due to cultural, religious, and political reasons, family planning is more controversial than many other public health issues.[1] Even the proponents of family

For peer review only - http://bmjopen.bmj.com/site/about/guidelines.xhtml

planning disagree with each other over what the primary aims should be. Some emphasize ecological concerns, specifically the effect of fertility declines on population structure, ecosystem, and economy. Others emphasize human rights concerns, promoting women's control over their own reproduction.[7]

Second, unlike other public health issues, such as reducing child mortality, the biomedical side of family planning is well-established, with proven methods to space and limit pregnancies. Where the successful implementation of family planning programs is concerned, it has been established that a key element is a political issue of obtaining support from and forming a broad coalition of elite groups.[1,7] This has proven successful in many countries, but remains elusive in some, especially in Sub-Saharan Africa.

The complexity of formulating effective strategies to promote women's and girls' access to family planning calls for closer coordination of resources and attention from all stakeholders. As noted by Kim and Ammann (2004), a clear consensus on targets and priorities is indispensable for all successful public projects in the modern era.[8]

During the past few years, two major family planning initiatives were launched. First, the London Summit on Family Planning in July 2012, was convened by the United Kingdom's Department for International Development (DFID) and the Bill & Melinda Gates Foundation (BMGF). At the Summit, leaders proposed adding 120 million female modern contraceptive users in the world's 69 poorest countries by 2020.[9] The second initiative, led by the United States Agency for International Development (USAID), proposed a target of satisfying 75% of the

For peer review only - http://bmjopen.bmj.com/site/about/guidelines.xhtml

demand for family planning with modern contraceptives by 2030.[10,11] This indicator of satisfied demand was subsequently adopted in the Sustainable Development Goals.[12] The percent satisfied demand is the proportion of women who use modern contraception divided by the total demand for family planning, which is defined by adding the percentage of married or in-union women aged 15-49 who are using any contraception to the percentage of women with unmet need. Unmet need refers to the proportion of women who want to stop or delay childbearing but are not using any method of contraception. Following Fabic et al., in the present study, we only consider the demand for FP among married or in-union women aged 15-49 years.[10]

FP2020 and 75% satisfied demand are two ambitious family planning initiatives. A recent assessment of FP2020 found that progress has been made with diverse country-level growth rates, but overall the initiative is below the proposed trajectory.[13] Given the scale of the initiatives and the number of partners involved in the family planning field, improved coordination, and a broader coalition is necessary to achieve the goals. The objective of this study is to assess the concordance of these two initiatives by estimating the implication of accomplishing one target on the other. A demonstration of their consistency, or the lack thereof, provides a better understanding of the proposed quantitative goals and helps to formulate collective strategies.

Methods

The contraceptive prevalence data are from the United Nations Development Programme (UNDP) survey-based estimates of the percentage of married or in-union women aged 15-49 using any modern contraceptive method.[14] The database includes estimates of modern

For peer review only - http://bmjopen.bmj.com/site/about/guidelines.xhtml

contraceptive prevalence rate (mCPR) and % satisfied demand collected from 466 surveys in 142 countries from 1986 to 2016. Among the 70 FP2020 focus countries (South Africa joined the FP2020 Initiative after the London Summit), three countries (Djibouti, Somalia, and Western Sahara) do not have any survey-based estimates of mCPR and % satisfied demand and therefore are excluded from the present study. In the end, our study is based on 67 FP2020 countries, with a focus on the 41 countries that made a commitment to the FP2020 Initiative (defined as commitment-making countries; see www.familyplanning2020.org for a full and up-to-date list; accessed on February 20, 2019).

The target measures discussed in this study are closely correlated by definition. Let P denote the total number of women aged 15-49 years, N denote the number of women who express a need for family planning, C denote the number of female modern contraceptive users, T denote the number of modern and traditional contraceptive users, U denote the number with unmet need for family planning. Then we have mCPR = C/P, % unmet need = U/P, and % met need (or satisfied demand) = C/N.

$$\%\, satisfied\ demand = \frac{C}{N} = \frac{C}{T+U} = \frac{mCPR}{CPR + \%unmet\ need}$$

An increase in C implies higher mCPR, but it does not necessarily increase % satisfied demand. The relationship between the indicators becomes complex in other scenarios, such as when more women express a need for family planning. This will decrease the % met need without affecting mCPR. The congruence, and lack of it, has been observed in FP2020 countries. From 2012 to 2017, the high growth of mCPR has driven a nine-percentage point increase in demand satisfied in Eastern and Southern Africa. During the same period, Central and West Africa experienced comparable mCPR growth, but that was accompanied by increasing levels of unmet need. These

For peer review only - http://bmjopen.bmj.com/site/about/guidelines.xhtml

are the results of a complex dynamic involving both fertility intentions and available family planning services. As a result, our subsequent empirical analyses will be based on probabilistic statistical regression rather than deterministic mathematical relationships.

Another complicating factor is that FP2020 counts all women, irrespective of their marital status, while the 75% target only covers married or in-union women. Although subsequent debates consider expanding the satisfied demand target to all women, no consensus has been reached, and therefore we will use the original statement of the 75% target. The difference in denominators will be dealt with in our statistical models.

The congruence between FP2020 and 75% satisfied demand targets requires a bi-directional assessment. We estimated the implications of achieving one of them on the other. Specifically, the study attempts to answer the following two questions: (1) how many additional users will be added following the 75% satisfied demand target; (2): what percentage of demand will be satisfied in 41 commitment-making countries assuming an annual increase of 1.4 percentage points from 2012 until 2030? Annual growth of 1.4% is the overall target proposed by the London Summit on Family Planning Metrics Group across all FP2020 focus countries [9]. Overall annual growth of 0.7 percentage points was observed across the world's 69 poorest countries before 2012. Brown et al. estimated that doubling the annual growth to 1.4 would add 120 million female modern contraceptive users by 2020. The target growth rate is considered an aspirational yet achievable goal assuming the resources and leadership around current family planning programs may be collectively mobilized. These two assessments are conducted separately, albeit employing a similar methodology (Figure 1).

For peer review only - http://bmjopen.bmj.com/site/about/guidelines.xhtml

<Figure 1 about here>

There are three steps to answer the first research question. The first step is to estimate the necessary married-woman mCPR to satisfy 75% demand with modern methods by 2030. Among the 41 commitment-making countries, five FP2020 commitment-making countries had already reached the 75% satisfied demand goal in their most recent surveys (Table 1). It is reasonable to assume that maintaining at least 75% satisfied demand by 2030 is the goal in those countries. We assume the mCPR and % satisfied demand will remain at their most recent observed level until 2030.

<Table 1 about here>

For the other 36 countries, the percentage of demand satisfied with modern methods is assumed to reach 75% in 2030. Then we employ the following country-level fixed effects longitudinal model to estimate the required mCPR for the assumed 75% satisfied demand.

$$y_{it} = \beta_0 + \beta_1 x_{it} + \beta_2 x_{it}^2 + \alpha_i + \varepsilon_{it} \quad (1)$$

where y_{it} denote the mCPR for country i in time t; x_{it} denotes the % satisfied demand for country i in time t; α_i denotes the time-invariant unobserved fixed effects for country i; ε_{it} denotes the error term. The mode is chosen from several options due to its best predictive performance. The model is first fitted using survey-based data compiled by the United Nations. The Least Squares Dummy Variable (LSDV) method is used in the model estimation [15]. This

For peer review only - http://bmjopen.bmj.com/site/about/guidelines.xhtml

approach explicitly provides the coefficients of the country dummy, which is required in predicting the mCPR for the assumed 75% satisfied demand in 2030. Then with the estimated coefficients and country-level fixed effects, we estimate mCPR for the assumed 75% satisfied demand.

The second step is to convert the married-woman mCPR estimated in step 1 to all-woman mCPR. Two hundred sixty-two DHS surveys based on samples of all women of reproductive ages were conducted from 1990 to 2016 in 85 countries. We use the following fixed effects longitudinal model to estimate all-woman mCPR from married mCPR

$$a_i = \theta_0 + \theta_1 m_i + v_i + \epsilon_{it} \quad (2)$$

where a_i and m_i denote the all-woman and married mCPR in survey i; v_i denotes region (SSA vs. non-SSA) level fixed effects. We use region level instead of country-level fixed effects because a model with country-level fixed effects cannot be used for prediction in FP2020 countries without a DHS survey.

In the third step, we assume all-woman mCPR will increase linearly from the level in the last survey to the level estimated for 2030 in step 2. Using the number of women of reproductive age obtained from World Population Prospects 2017, we calculate the number of female modern contraceptive users in the 67 FP2020 focus countries.[16]

For peer review only - http://bmjopen.bmj.com/site/about/guidelines.xhtml

The second research question is answered similarly in three steps (Figure 1). We first estimate the baseline, i.e., all-woman mCPR in 2012. Our principle is to rely on the survey-based estimates as much as possible. As mentioned above, 5 of the 41 FP2020 commitment-making countries had already reached the 75% satisfied demand goal in their most recent surveys, and therefore are excluded from this investigation. Among the other 36 commitment-making countries, 10 conducted a survey in 2012. For those 19 countries that have conducted surveys both before and after 2012, we use the two surveys before and after 2012 to linearly interpolate the mCPR for 2012. For the other 7 countries that only have surveys conducted before 2012, we used the last survey-based estimate for 2012.

Then we impose a 1.4% annual increase in all-woman mCPR from 2012 until 2030. Finally, we predict the % satisfied demand associated with the calculated levels of all-woman mCPR for 2012-2030 based on a fixed effects longitudinal model similar to Equation (1), but moving % satisfied demand to the left-hand side and including mCPR and its squared term in the right-hand side.

Patient and Public Involvement

The study does not involve patients or the public.

Results

All three fixed effects longitudinal models fit the data quite well, indicating excellent predictive performance (Table 2). Using 466 survey-based estimates, the adjusted R-squared of the model regressing married-woman mCPR on % satisfied demand and a country dummy is above 0.98,

For peer review only - http://bmjopen.bmj.com/site/about/guidelines.xhtml

For peer review only

meaning that less than 2% of the variations in married-woman mCPR cannot be explained by the model (Model 1). As a result, the estimated married-woman mCPR based on the assumed 75% satisfied demand should be highly accurate and reliable. The adjusted R-squared of 0.97 in Model 2 also indicates accurate conversion from married- to all-woman mCPR. Model 3 that regresses % satisfied demand on married-woman mCPR also performed well (adjusted R-squared 0.97).

<Table 2 about here>

Achieving the 75% satisfied demand by 2030 goal means a gain of approximately 82 million additional users in these 67 FP2020 countries from 2012 to 2020, which is about 68% of the 120 million proposed by the FP2020 Initiative (Table 3). From 2012 to 2020, these 41 commitment-making countries will contribute 74 million additional users while these 26 non-commitment-making FP2020 countries contribute 8 million. If the 67 countries continue the mCPR growth rate implied by the 75% satisfied demand initiative, the goal of adding 120 million female modern contraceptive users will be achieved in early 2023 (Figure 2). By 2030, there will be 184 and 21 million additional users in commitment-making and non-commitment-making countries, respectively, making a total number of 206 additional modern contraceptive users in these 67 FP2020 countries.

<Table 3 about here>

Five of the 41 FP2020 commitment-making countries (three in sub-Saharan Africa)have already satisfied 75% or more of the contraceptive demand, according to their last survey. Among the other 36 commitment-making countries, only four additional countries (Bangladesh, India,

For peer review only - http://bmjopen.bmj.com/site/about/guidelines.xhtml

Malawi, and Vietnam) will reach that target by 2020, following FP2020's proposed 1.4% annual increase in mCPR (Table 4). Another eight countries (Ethiopia, Laos, Madagascar, Nepal, Rwanda, Solomon Islands, Tanzania, and Zambia) will do so by 2030. Disaggregated by region, the situation is more challenging in sub-Saharan Africa, where only one (Malawi) of the 26 commitment-making countries will reach the 75% target by 2020, and another five countries (Ethiopia, Madagascar, Rwanda, Tanzania, and Zambia) will do so by 2030. Adding those three countries that had already reached the target in their most recent surveys, less than one third (9) of the 29 commitment-making countries in this region will satisfy 75% demand for family planning by 2030. In the other regions, five countries will achieve the target by 2020, and another three will do so by 2030. Those eight target-achieving countries represent two-thirds of the 12 non-SSA commitment-making countries.

In sum, assuming FP2020's proposed annual growth rate in mCPR, the % satisfied will reach 75% in less than half (17) of the 41 FP2020 commitment-making countries

<Table 4 about here>

Discussion

The contribution of this study is an improved understanding of the concordance of two global family planning initiatives: FP2020's adding 120 female modern contraceptive users by 2020 in 69 of the world's poorest countries and USAID's satisfying 75% demand for family planning with modern contraceptives. Our results show that the two initiatives move towards the same goal of promoting access to family planning for women and girls. Overall, both the 75% satisfied demand and the FP2020 goal are ambitious. Achieving the 75% satisfied demand goal by 2030

For peer review only

For peer review only - http://bmjopen.bmj.com/site/about/guidelines.xhtml

implies that 82 million or 68% of the 120-million target users will be added by 2020 in 67 FP2020 focus countries. The target of 120 million will be achieved by 2023, only three years later than the FP2020 deadline. On the other hand, achieving a 1.4% annual increase in all-woman mCPR will enable only 17 of the 41 commitment-making countries to attain the goal of 75% satisfied demand by 2030. The overall assessment should not mask the across-country variations. In some countries it is more plausible to achieve the FP2020's proposed annual increase of 1.4 percentage points than satisfying the 75% demand by 2030. Some other countries, however, have satisfied 75% demand or will do so by 2030 with an annual mCPR increase below 1.4 percentage points.

Capitalizing the shared goals, the demonstrated concordance may facilitate building a broad coalition to promote family planning in the developing world. These two initiatives represent the objectives of two major donors to family planning.[17] The % satisfied demand has also been adopted as an indicator of the Sustainable Development Goals. Due to their different features and advantages, mCPR (and number of users) and % satisfied demand will continue coexisting in the international agenda for family planning. Despite their theoretical correlation, the empirical relation between the two indicators depends on other context-specific factors, such as demand generation and changes in fertility desire. As a result, an assessment of the empirical correlation between the two indicators has sustaining policy implications. A consensus goal is critical to building a broad coalition to collectively and effectively mobilize financial and political resources and capture global attention.

For peer review only - http://bmjopen.bmj.com/site/about/guidelines.xhtml

The simulated implications of achieving one target on the other have several policy implications, which are urgently needed as donors and stakeholders are debating about the post-FP2020 plan. First, multiple measures will continue coexisting in international family planning. The FP2020 Core Group of which the Bill and Melinda Gates Foundation, United Kingdom's Department for International Development (DFID), USAID and United Nations Population Fund (UNFPA) are active may renew their commitment to adding female modern contraceptive users beyond FP2020 to the FP2030 deadline. The % satisfied demand has been adopted as an indicator, SDG 3.7.1. The 75% benchmark is being used as a proxy for the minimum definition of "universal access to reproductive health" in terms of contraceptive use (SDG 3). Methodologically, our models for assessing the congruence of the two measures could be replicated as the FP2020 movement sets its goals for FP2030.

Second, our exercise sheds light on the choice between aspirational and realistic target-setting approaches. The findings show that 75% satisfied demand can be viewed in three settings: 1) countries who have already achieved the goal but whose plans involve increasing the percentage higher than the 75% benchmark (e.g., Indonesia, Myanmar, Kenya, South Africa), Zimbabwe); 2) countries which are projected to likely reach the goal by 2020 and 2030; and 3) countries which will remain below the goal (24 of 41 commitment-making countries). With only one year left before its deadline, FP2020 has contributed to the mobilization of global resources for family planning and has shown progress against the goal but not at the trajectory to reach 120 million more women and girls by 2020.

For peer review only - http://bmjopen.bmj.com/site/about/guidelines.xhtml

The third policy implication is for the choice between global and national targets. All countries in our exercise belong to low-income countries, but they still demonstrate massive diversity in terms of mCPR, desired and realized fertility, and population age structure. When setting targets in the future, donors and stakeholders need to strike a balance between simplification (global target as in FP2020) and customization (country-specific targets as in 75% satisfied demand).

The last policy implication is on SDG. Although % satisfied demand has been adopted as an indicator (SDG 3.7.1), it has not been associated with quantitative goals. The same situation occurred to Target 5b of Millennium Development Goals (MDGs): "Achieve, by 2015, universal access to reproductive health". Several studies argued that clear, measurable goals could be a focal point for coalescing political support for action.[9] Reflecting on the lag in substantively integrating family planning into the MDGs, FP2020 proposed a quantifiable target of adding 120 million female modern contraceptive users by 2020. Adopting the target of 75% satisfied demand in SDG may help mobilize and guide resource allocation and provide a benchmark for program advocacy. Per our simulation results, the target is achievable in certain countries and aspirational in others.

The study is not without limitations. First, despite the highly satisfactory model fit, our regressions could be theoretically improved by including other factors such as calendar time. We did not include year as a covariate because its coefficient reflects not only temporal effects but also the changing composition of countries in the database. For example, the earliest DHS surveys were mostly in Africa, while Asia was added later. So, the absence of calendar time in the model is a limitation with the database rather than our methodology. Since we are mainly

For peer review only - http://bmjopen.bmj.com/site/about/guidelines.xhtml

interested in the predictive performance of the model, measured by the adjusted R-squared, and adding year as a covariate changed the adjusted R-squared by less than 1 percentage point, our final model did not consider calendar time. The second limitation is the linear assumption on mCPR growth. Other growth curves (such as S-shaped or logistic) may be more accurate in many countries. The 67 FP2020 countries are in different stages of mCPR growth, some experiencing a convex trajectory and some a concave trajectory. Fully accounting for country-specific curves will likely make the statistical models much more complex and less robust. We believe a linear trajectory provides an acceptable approximation for the mixture of convex and concave trajectories. Consequently, the global estimates presented in the study may not be substantially affected by the assumed linearity.

As repeatedly emphasized in the London Summit document, setting a quantitative target should not cause concern among those firmly committed to sexual and reproductive health and rights because all interventions will have women's rights at the center of their implementation efforts. Our assessment in this study of the congruence of major, articulated family planning initiatives aims to unite international communities into collective actions that secure women's and girls' access to effective contraceptive methods.

Author contributions: QL, SA, and JR devised the study and wrote the article. QL compiled the data and led the statistical modeling and analysis.

Disclosure statement: No potential conflict of interest was reported by the authors.

Ethics and consent: This study only uses publicly available secondary data and does not involve any participants. Therefore, ethical approval and participant consent are not required.

For peer review only

For peer review only - http://bmjopen.bmj.com/site/about/guidelines.xhtml

Data sharing statement: All data in this paper were obtained from published studies. No additional data are available from the authors.

Funding: Bill and Melinda Gates Foundation

References

1. Cleland J, Bernstein S, Ezeh A, Faundes A, Glasier A, Innis J. Family planning: the unfinished agenda. *The Lancet* 2006; **368**(9549): 1810-27.
2. Ahmed S, Li Q, Liu L, Tsui AO. Maternal deaths averted by contraceptive use: an analysis of 172 countries. *The Lancet* 2012; **380**(9837): 111-25.
3. Cleland J, Conde-Agudelo A, Peterson H, Ross J, Tsui A. Contraception and health. *Lancet (London, England)* 2012; **380**(9837): 149-56.
4. Canning D, Schultz TP. The economic consequences of reproductive health and family planning. *Lancet (London, England)* 2012; **380**(9837): 165-71.
5. Cohen SA. London summit puts family planning back on the agenda, offers new lease on life for millions of women and girls. *Guttmacher Policy Review* 2012; **15**(3): 20-4.
6. Ezeh AC, Bongaarts J, Mberu B. Global population trends and policy options. *Lancet (London, England)* 2012; **380**(9837): 142-8.
7. Shiffman J, Quissell K. Family planning: a political issue. *Lancet (London, England)* 2012; **380**(9837): 181-5.
8. Kim JY, Ammann A. Is the “3 by 5” Initiative the Best Approach to Tackling the HIV Pandemic? *PLoS Medicine* 2004; **1**(2): e37.
9. Brown W, Druce N, Bunting J, et al. Developing the "120 by 20" goal for the Global FP2020 Initiative. *Stud Fam Plann* 2014; **45**(1): 73-84.
10. Fabic MS, Choi Y, Bongaarts J, et al. Meeting demand for family planning within a generation: the post-2015 agenda. *Lancet (London, England)* 2015; **385**(9981): 1928-31.
11. Choi Y, Short Fabic M. Monitoring Progress in Equality for the Sustainable Development Goals: A Case Study of Meeting Demand for Family Planning. *Global health, science and practice* 2018; **6**(2): 390-401.
12. United Nations General Assembly. Sustainable Development Goals 3. https://sustainabledevelopment.un.org/sdg3 (accessed August 9 2019).
13. Cahill N, Sonneveldt E, Stover J, et al. Modern contraceptive use, unmet need, and demand satisfied among women of reproductive age who are married or in a union in the focus countries of the Family Planning 2020 initiative: a systematic analysis using the Family Planning Estimation Tool. *Lancet (London, England)* 2017.
14. United Nations DoEaSA, Population Division (2017). World Contraceptive Use 2017 (POP/DB/CP/Rev2017).
15. Greene WH. Econometric analysis: Prentice Hall; 2011.
16. United Nations DoEaSA, Population Division, W. World Population Prospects: The 2017 Revision, Key Findings and Advance Tables. Working Paper No. ESA/P/WP/248. 2017.
17. IHME. Financing Global Health 2014: Shifts in Funding as the MDG Era Closes. Seattle, WA: IHME; 2015.

For peer review only - http://bmjopen.bmj.com/site/about/guidelines.xhtml

Figure 1: Analytical flowchart for the two research questions

Figure 2: Number of additional female modern contraceptive users in 41 commitment-making and 26 non-commitment-making countries assuming the trajectory of satisfying 75% demand by 2030

For peer review only

Table 1: Modern contraceptive prevalence rate (mCPR) in 5 commitment-making countries where % demand satisfied exceeded 75% in the last survey

Country	Region	Survey date	mCPR	% demand satisfied
Myanmar	Non-SSA	2015-16	51.3	75.0
Kenya	SSA	2015	62.6	76.2
Indonesia	Non-SSA	2015-16	59.5	78.8
South Africa	SSA	2003-04	59.8	81.1
Zimbabwe	SSA	2015	65.8	85.2

Notes: SSA denotes sub-Sahara Africa; non-SSA includes all other regions

For peer review only

Table 2: Goodness of fit of the fixed effects longitudinal models

	Model 1	Model 2	Model 3
**Outcome**	Married mCPR	All-woman mCPR	% satisfied demand
**Covariates**	% satisfied demand	Married mCPR	Married mCPR
	(% satisfied demand)^2		(married mCPR)^2
**Fixed effects**	Country level	Region (SSA; non-SSA) level	Country level
**Sample size**	466	262	466
**R-squared**	0.98	0.91	0.97

For peer review only

For peer review only - http://bmjopen.bmj.com/site/about/guidelines.xhtml

Table 3: Modern contraceptive prevalence rate (mCPR), modern contraceptive users (thousand), and added users since 2012 (thousand) in 41 commitment-making and 26 non-commitment-making countries under FP2020: assuming the achievement of 75% satisfied demand by 2030

Country	2012		2020			2030		
	mCPR	Users	mCPR	Users	Added users	mCPR	Users	Added users
**Commitment-making countries (41)**								
Afghanistan	10.4	691	22.7	2,065	1,374	38.1	4,645	3,954
Bangladesh	41.2	17,800	42.4	20,200	2,366	43.8	22,200	4,369
Benin	7.0	158	22.0	636	478	40.9	1,570	1,412
Burkina Faso	13.5	515	25.5	1,249	734	40.5	2,704	2,189
Burundi	16.7	371	27.5	768	397	41.0	1,599	1,229
Cameroon	14.2	708	25.8	1,621	913	40.3	3,362	2,655
Chad	0	0	17.2	651	651	39.3	2,052	2,052
Côte d'Ivoire	11.5	566	24.2	1,509	943	39.9	3,277	2,711
DR Congo	3.9	594	20.3	4,066	3,472	40.8	11,600	11,000
Ethiopia	26.2	5,677	32.6	9,285	3,608	40.5	15,100	9,398
Ghana	14.3	935	26.9	2,093	1,158	42.6	4,080	3,145
Guinea	4.8	124	20.1	655	531	39.2	1,711	1,588
Haiti	24.5	663	32.9	1,009	346	43.3	1,493	829
India	35.7	115,000	38.5	138,000	22,600	41.9	162,000	46,700
Indonesia	45.7	31,000	45.7	32,900	1,904	45.7	34,600	3,521
Kenya	47.1	5,076	47.7	6,592	1,516	48.5	8,575	3,499
Laos	33.4	570	38.1	742	171	44.1	972	401
Liberia	14.2	139	25.9	318	179	40.6	649	510
Madagascar	26.2	1,395	32.8	2,246	851	41.0	3,674	2,280
Malawi	45.4	1,696	44.7	2,218	522	44.0	2,984	1,289
Mali	7.6	268	21.2	962	694	38.1	2,427	2,160
Mauritania	7.6	70	22.2	259	189	40.5	610	540

For peer review only - http://bmjopen.bmj.com/site/about/guidelines.xhtml

Mozambique	11.3	676	23.3	1,792	1,115	38.2	4,002	3,325
Myanmar	39.5	5,503	39.5	5,956	453	39.5	6,197	694
Nepal	34.0	2,504	38.1	3,293	789	43.4	4,078	1,574
Niger	5.2	192	19.2	971	778	36.6	2,781	2,588
Nigeria	6.2	2,364	20.5	9,773	7,409	38.5	24,200	21,900
Pakistan	20.0	8,935	29.5	15,400	6,475	41.3	26,100	17,100
Philippines	28.1	7,022	35.8	10,100	3,100	45.5	14,700	7,664
Rwanda	36.1	974	39.2	1,326	353	43.1	1,880	906
Senegal	12.8	429	24.7	1,040	611	39.6	2,217	1,787
Sierra Leone	11.6	188	23.7	479	291	38.8	997	809
Solomon Islands	24.1	33	28.0	45	13	32.7	64	31
South Africa	46.4	6,715	46.4	7,366	651	46.4	8,067	1,352
South Sudan	6.3	160	21.2	697	537	39.9	1,715	1,555
Togo	11.8	195	25.1	516	322	41.7	1,109	915
Uganda	20.5	1,652	30.7	3,355	1,703	43.4	6,715	5,062
Tanzania	21.8	2,483	30.1	4,459	1,976	40.4	8,243	5,760
Viet Nam	43.4	11,200	45.8	11,900	686	48.8	12,600	1,401
Zambia	34.3	1,176	38.0	1,731	555	42.7	2,627	1,451
Zimbabwe	50.9	1,944	50.9	2,366	422	50.9	3,010	1,066
**Subtotal**		**238,350**		**312,597**	**73,836**		**423,176**	**184,372**
**Non-commitment-making countries (26)**								
Bhutan	50.2	101	50.2	114	14	50.2	124	23
Bolivia	30.5	788	37.6	1,129	341	46.6	1,590	801
Cambodia	28.5	1,159	35.1	1,581	422	43.3	2,281	1,121
Central African Republic	12.7	133	24.7	292	158	39.7	627	493
Comoros	12.0	21	25.3	54	33	41.9	114	92
Congo	10.9	120	24.9	334	214	42.3	755	635

For peer review only

Egypt	43.8	9,961	43.8	11,200	1,256	43.8	13,200	3,262
Eritrea	9.9	108	23.1	311	203	39.6	704	595
Gambia	5.6	24	19.8	109	85	37.6	282	258
Guinea-Bissau	10.2	41	18.1	90	49	27.9	180	139
Honduras	49.0	1,104	49.0	1,319	215	49.0	1,495	391
Iraq	28.5	2,270	33.5	3,367	1,097	39.6	5,157	2,887
Kyrgyzstan	26.3	401	32.1	501	100	39.4	703	301
Lesotho	46.4	251	46.4	290	39	46.4	337	86
Mongolia	36.9	305	39.5	331	26	42.8	392	87
Nicaragua	59.2	960	59.2	1,053	93	59.2	1,119	159
North Korea	58.5	3,899	58.5	3,800	-100	58.5	3,654	-246
Palestine	33.1	348	37.0	490	143	41.9	717	369
Papua New Guinea	24.6	449	32.5	717	267	42.3	1,130	680
Sao Tome and Principe	27.3	12	36.2	19	7	47.4	32	20
Sri Lanka	42.1	2,251	44.6	2,374	123	47.6	2,473	222
Sudan	6.5	552	21.1	2,240	1,688	39.3	5,425	4,873
Tajikistan	20.3	428	28.0	670	242	37.5	1,086	657
Timor-Leste	18.2	46	24.0	74	28	31.1	127	81
Uzbekistan	39.9	3,334	40.1	3,597	263	40.3	3,964	630
Yemen	21.8	1,326	30.3	2,339	1,013	41.0	4,079	2,753
**Subtotal**		**30,395**		**38,397**	**8,019**		**51,744**	**21,372**
**Total (67 focus countries)**		**268,745**		**350,995**	**81,855**		**474,920**	**205,744**

Notes: the predicted mCPR for Chad 2012 was rounded to 0; the columns may add up exactly because our statistical models used exact numbers while results are presented in thousands.

For peer review only

Table 4: Modern contraceptive prevalence rate (mCPR) and % demand satisfied (%SD) in 2012, 2020 and 2030 in 36 commitment-making countries: assuming the achievement of FP2020 and extending its mCPR trajectories to 2030

Country	2020		2030	
	mCPR	%SD	mCPR	%SD
**Sub-Sahara Africa(26)**				
Benin	19.1	33.7	33.1	52.0
Burkina Faso	27.4	47.1	41.4	63.5
Burundi	28.9	46.5	42.9	62.5
Cameroon	27.8	48.3	41.8	64.5
Chad	15.9	31.4	29.9	50.5
Côte d'Ivoire	23.7	42.6	37.7	59.8
DR Congo	18.8	33.5	32.8	51.9
Ethiopia	40.9	63.6	54.9	**76.7**
Ghana	32.8	50.5	46.8	65.6
Guinea	15.8	31.8	29.8	50.9
Liberia	28.9	49.0	42.9	65.0
Madagascar	40.4	61.4	54.4	**74.7**
Malawi	62.1	**77.7**	76.1	**85.8**
Mali	21.1	42.6	35.1	60.5
Mauritania	22.7	40.8	36.7	58.3
Mozambique	22.5	45.6	36.5	63.1
Niger	23.4	49.9	37.4	67.2
Nigeria	22.0	44.7	36.0	62.3
Rwanda	56.8	73.3	70.8	**82.6**
Senegal	27.3	48.6	41.3	65.0
Sierra Leone	24.9	47.9	38.9	64.9
South Sudan	12.9	23.8	26.9	43.6
Togo	26.9	42.8	40.9	59.3
Uganda	37.0	54.6	51.0	68.7
Tanzania	40.5	63.8	54.5	**77.0**
Zambia	53.1	72.0	67.1	**82.2**
**Other regions (10)**				
Afghanistan	31.0	56.9	45.0	72.4
Bangladesh	70.5	**83.1**	84.5	**89.2**
Haiti	42.5	59.7	56.5	72.5
India	59.1	**78.0**	73.1	**86.8**
Laos	53.9	70.1	67.9	**80.2**

For peer review only

For peer review only - http://bmjopen.bmj.com/site/about/guidelines.xhtml

Nepal	54.4	72.1	68.4	**82.0**
Pakistan	37.3	57.7	51.3	71.6
Philippines	48.3	62.7	62.3	74.0
Solomon Islands	38.6	73.3	52.6	**87.0**
Viet Nam	69.7	**77.3**	83.7	**83.6**

Notes: bold indicates reaching the target of satisfying 75% demand for family planning; Madagascar's 74.7% in 2030 can be rounded to 75%.

For peer review only

For peer review only - http://bmjopen.bmj.com/site/about/guidelines.xhtml

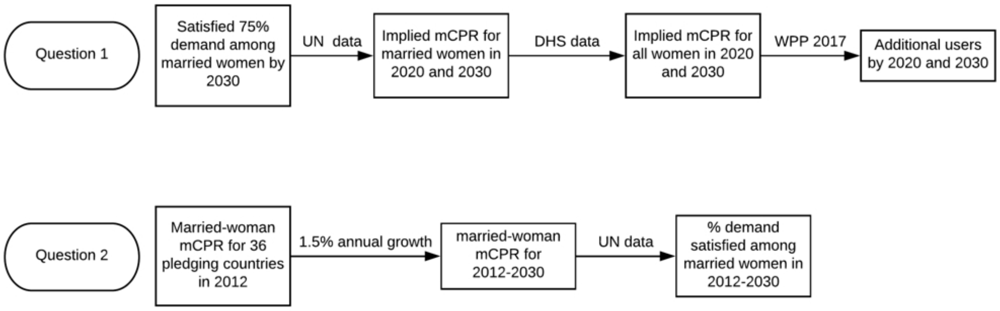

Figure 1: Analytical flowchart for the two research questions

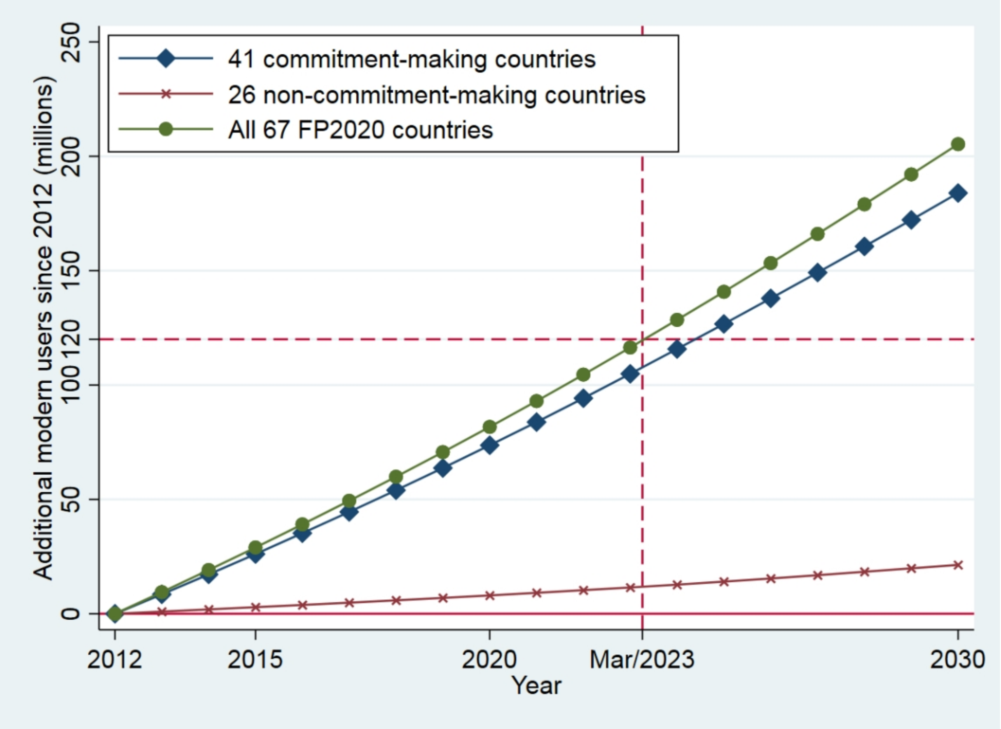

Figure 2: Number of additional modern contraceptive users in 41 pledging and 26 non-pledging countries assuming the trajectory of satisfying 75% demand by 2030

STROBE Statement—checklist of items that should be included in reports of observational studies

	Item No.	Recommendation	Page No.
**Title and abstract**	1	(*a*) Indicate the study's design with a commonly used term in the title or the abstract	1
		(*b*) Provide in the abstract an informative and balanced summary of what was done and what was found	2-3
**Introduction**			
Background/rationale	2	Explain the scientific background and rationale for the investigation being reported	4-6
Objectives	3	State specific objectives, including any prespecified hypotheses	6
**Methods**			
Study design	4	Present key elements of study design early in the paper	6
Setting	5	Describe the setting, locations, and relevant dates, including periods of recruitment, exposure, follow-up, and data collection	6-7
Participants	6	(*a*) *Cohort study*—Give the eligibility criteria, and the sources and methods of selection of participants. Describe methods of follow-up<br>*Case-control study*—Give the eligibility criteria, and the sources and methods of case ascertainment and control selection. Give the rationale for the choice of cases and controls<br>*Cross-sectional study*—Give the eligibility criteria, and the sources and methods of selection of participants	n/a
		(*b*) *Cohort study*—For matched studies, give matching criteria and number of exposed and unexposed<br>*Case-control study*—For matched studies, give matching criteria and the number of controls per case	n/a
Variables	7	Clearly define all outcomes, exposures, predictors, potential confounders, and effect modifiers. Give diagnostic criteria, if applicable	6-8
Data sources/ measurement	8*	For each variable of interest, give sources of data and details of methods of assessment (measurement). Describe comparability of assessment methods if there is more than one group	*6-11*
Bias	9	Describe any efforts to address potential sources of bias	6-11
Study size	10	Explain how the study size was arrived at	6

Continued on next page

For peer review only - http://bmjopen.bmj.com/site/about/guidelines.xhtml

For peer review only

Quantitative variables	11	Explain how quantitative variables were handled in the analyses. If applicable, describe which groupings were chosen and why	6-8
Statistical methods	12	(*a*) Describe all statistical methods, including those used to control for confounding	6-11
		(*b*) Describe any methods used to examine subgroups and interactions	n/a
		(*c*) Explain how missing data were addressed	n/a
		(*d*) *Cohort study*—If applicable, explain how loss to follow-up was addressed<br>*Case-control study*—If applicable, explain how matching of cases and controls was addressed<br>*Cross-sectional study*—If applicable, describe analytical methods taking account of sampling strategy	n/a
		(*e*) Describe any sensitivity analyses	n/a
**Results**			
Participants	13*	(a) Report numbers of individuals at each stage of study—eg numbers potentially eligible, examined for eligibility, confirmed eligible, included in the study, completing follow-up, and analysed	n/a
		(b) Give reasons for non-participation at each stage	n/a
		(c) Consider use of a flow diagram	n/a
Descriptive data	14*	(a) Give characteristics of study participants (eg demographic, clinical, social) and information on exposures and potential confounders	n/a
		(b) Indicate number of participants with missing data for each variable of interest	n/a
		(c) *Cohort study*—Summarise follow-up time (eg, average and total amount)	n/a
Outcome data	15*	*Cohort study*—Report numbers of outcome events or summary measures over time	n/a
		Case-control study—Report numbers in each exposure category, or summary measures of exposure	n/a
		Cross-sectional study—Report numbers of outcome events or summary measures	n/a
Main results	16	(*a*) Give unadjusted estimates and, if applicable, confounder-adjusted estimates and their precision (eg, 95% confidence interval). Make clear which confounders were adjusted for and why they were included	11-13
		(*b*) Report category boundaries when continuous variables were categorized	n/a
		(*c*) If relevant, consider translating estimates of relative risk into absolute risk for a meaningful time period	n/a

Continued on next page

For peer review only - http://bmjopen.bmj.com/site/about/guidelines.xhtml

Other analyses	17	Report other analyses done—eg analyses of subgroups and interactions, and sensitivity analyses	n/a
**Discussion**			
Key results	18	Summarise key results with reference to study objectives	11-13
Limitations	19	Discuss limitations of the study, taking into account sources of potential bias or imprecision. Discuss both direction and magnitude of any potential bias	16-17
Interpretation	20	Give a cautious overall interpretation of results considering objectives, limitations, multiplicity of analyses, results from similar studies, and other relevant evidence	17
Generalisability	21	Discuss the generalisability (external validity) of the study results	17
**Other information**			
Funding	22	Give the source of funding and the role of the funders for the present study and, if applicable, for the original study on which the present article is based	18

*Give information separately for cases and controls in case-control studies and, if applicable, for exposed and unexposed groups in cohort and cross-sectional studies.

Note: An Explanation and Elaboration article discusses each checklist item and gives methodological background and published examples of transparent reporting. The STROBE checklist is best used in conjunction with this article (freely available on the Web sites of PLoS Medicine at http://www.plosmedicine.org/, Annals of Internal Medicine at http://www.annals.org/, and Epidemiology at http://www.epidem.com/). Information on the STROBE Initiative is available at www.strobe-statement.org.

For peer review only
